# The pro- and anti-tumor roles of mesenchymal stem cells toward BRCA1-IRIS-overexpressing TNBC cells

**DOI:** 10.1186/s13058-019-1131-2

**Published:** 2019-04-24

**Authors:** Daniel Ryan, Bibbin T. Paul, Jim Koziol, Wael M. ElShamy

**Affiliations:** 1 Breast Cancer Program, San Diego Biomedical Research Institute, 10865 Road to Cure, Suite 100, San Diego, CA 92121 USA; 2 Department of Molecular Biology and Biophysics, University of Connecticut Health Center, Farmington, CT USA; 3 Department of Molecular and Experimental Medicine, The Scripps Research Institute, La Jolla, CA USA

**Keywords:** BRCA1-IRIS, Triple-negative breast cancer, Metastasis, Mesenchymal stem cells, IL-6, Prostaglandin E_2_, STAT3

## Abstract

**Background:**

To evaluate the cross-talk between BRCA1-IRIS (IRIS)-overexpressing (IRISOE) TNBC cells and tumor-resident mesenchymal stem cells (MSCs) that triggers the aggressiveness or elimination of IRISOE TNBC tumors.

**Methods:**

We analyzed the effect of silencing or inactivating IRIS on the bi-directional interaction between IRISOE TNBC cells and MSCs on tumor formation and progression. We analyzed the downstream signaling in MSCs induced by IL-6 secreted from IRISOE TNBC cells. We compared the effect of MSCs on the formation and progression of IRIS-proficient and deficient-TNBC cells/tumors using in vitro and in vivo models. Finally, we analyzed the association between IL-6, PTGER2, and PTGER4 overexpression and breast cancer subtype; hormone receptor status; and distant metastasis-free or overall survival.

**Results:**

We show high-level IL-6 secreted from IRISOE TNBC cells that enhances expression of its receptor (IL-6R) in MSCs, their proliferation, and migration toward IRISOE, in vitro, and recruitment into IRISOE TNBC tumors, in vivo. In serum-free medium, recombinant IL-6 and the IL-6-rich IRISOE TNBC cell condition media (CM) decreased STAT3^Y705^ phosphorylation (p-STAT3^Y705^) in MSCs. Inhibiting IRIS expression or activity prolonged STAT3^Y705^ phosphorylation in MSCs. The interaction with IRISOE TNBC cells skewed MSC differentiation toward prostaglandin E_2_ (PGE_2_)-secreting pro-aggressiveness cancer-associated fibroblasts (CAFs). Accordingly, co-injecting human or mouse MSCs with IRISOE TNBC tumor cells promoted the formation of aggressive mammary tumors, high circulating IL-6 and PGE_2_ levels, and reduced overall survival. In contrast, IRIS-silenced or inactivated cells showed reduced tumor formation ability, limited MSC recruitment into tumors, reduced circulating IL-6 and PGE_2_ levels, and prolonged overall survival. A positive correlation between IL-6, PTGER2, and PTGER4 expression and basal phenotype; ER-negativity; distant metastasis-free and overall survival in basal; or BRCA^mutant^ carriers was observed. Finally, the bi-directional interaction with MSCs triggered death rather than growth of IRIS-silenced TNBC cells, in vitro and in vivo.

**Conclusions:**

The IL-6/PGE_2_-positive feedback loop between IRISOE TNBC tumor cells and MSCs enhances tumor aggressiveness. Inhibiting IRIS expression limits TNBC tumor growth and progression through an MSC-induced death of IRIS-silenced/inactivated TNBC cells.

**Electronic supplementary material:**

The online version of this article (10.1186/s13058-019-1131-2) contains supplementary material, which is available to authorized users.

## Background

The microenvironment plays an important role in promoting breast cancer progression [1]. Triple-negative breast cancer (TNBC) is the most aggressive breast cancer subtype, yet it is the one lacking effective therapy. TNBCs feature a unique microenvironment, distinct from that of other subtypes, especially the less aggressive luminal A subtype [2]. Bi-directional interactions with microenvironment entities—such as immune cells, cancer-associated fibroblasts (CAFs), mesenchymal stem cells (MSCs), or tumor-associated macrophages (TAMs)—exacerbate TNBC tumor cells’ aggressiveness [3–7]. Certain soluble factors secreted from tumor or microenvironment cells synergistically impede an effective anti-tumor response and promote breast cancer progression and metastasis [2].

MSCs are a heterogeneous population of non-hematopoietic progenitors predominantly found in the bone marrow [8]. MSCs are capable of self-renewal and differentiation into several cell types (e.g., chondrocytes, adipocytes, osteocytes, and fibrocytes [6, 9]). Under pathological conditions such as tissue injury or cancer, MSCs are recruited by pro-inflammatory factors, such as interleukin-1β and IL-6 secreted by injured cells or tumor cells to aid in the repair [10, 11]. MSCs also home toward the hypoxic tumor microenvironment, in vivo [12].

BRCA1-IRIS (*aka* IRIS, for ***I***n-frame ***R***eading of BRCA1 ***I****ntron* 11 ***S***plice variant) is an oncogene produced by the alternative usage of the *BRCA1* locus rather than the alternative splicing of the *BRCA1 mRNA* [13]. While IRIS expression is high in all breast cancer subtypes, TNBCs express the highest level [14]. Deliberate IRIS overexpression (IRISOE) in normal mammary epithelial cells or luminal A/ER^+^ cells converts them into genuine TNBC cells expressing basal biomarkers, epithelial-to-mesenchymal (EMT) inducers, and stemness enforcers, but lacking expression of ERα and BRCA1 proteins, in vitro and in vivo [15, 16]. Moreover, while normal mammary epithelial cells (HME) expressing mutant Ras^V12^ or overexpressing IRIS develop mammary tumors in SCID mice, unlike Ras^V12^-driven tumors that showed luminal phenotype and expressed ERα and BRCA1 proteins [14, 17], IRISOE-driven tumors contained a large necrotic/hypoxic core [14], showed mesenchymal phenotype and were more aggressive. This data adds support to our recently published hypothesis that a harsh microenvironment, such as necrosis/hypoxia/inflammation within TNBC, generates an aggressiveness niche in which metastatic precursors are born.

Indeed, under the hypoxic or inflamed conditions within the aggressiveness niche, IRISOE TNBC tumor cells secrete high levels of IL-1β, which serve to activate and attract MSCs [11]. Activated MSCs then secrete other inflammatory cytokines, such as CXCL1 [18–20], which signals through CXCR2 expressed on IRISOE TNBC cancer cells to increase their dissemination ability and poor patient prognosis, chemo-resistance, and metastasis [18, 21]. Therapeutic targeting of the IL-1β/IL-1R or the CXCL1/CXCR2 circuits in an adjuvant setting circumvents chemotherapy resistance in breast cancer patients [18, 21], and the pre-clinical model of IRISOE TNBC tumor [12].

The role of IL-6 in breast cancer growth and progression is complicated. IL-6 produced by the microenvironment within TNBC tumors enhances tumor growth and metastasis [22–24]. There is a lack of information about the effect of IL-6 produced by TNBC tumor cells on the microenvironment entities, such as MSCs. Here, we report that IL-6 secreted from IRISOE TNBC cells activates STAT3, AKT, and ERK/MAPK signaling in MSCs in a paracrine fashion to enhance their proliferation, migration, and survival. Inhibiting IL-6 signaling utilizing neutralizing antibodies attenuated MSC migration. One of the major purposes of the current study was to demonstrate that hypoxic IRISOE TNBC tumor cells recruit MSCs and activate them to promote their own aggressiveness. Another major purpose was to show that resident MSCs can have an anti-tumor role in which they are able to eliminate IRIS-silenced/inactivated TNBC tumors.

## Methods

### Cell culture

All commercially available cell lines were obtained from ATCC and maintained as previously described [17]. The doxycycline (Dox)-inducible IRISOE cell lines’ (IRISOE1-5) generation and maintenance were described earlier [13, 25]. These cell lines develop into primary (1°) orthotopic IRISOE mammary tumors when injected in SCID mice and the mice given Dox-supplemented drinking water (naïve HME do not survive in vivo [14, 17]). Three cell lines—“IRIS291, IRIS292, and IRIS293”—were developed from these resected 1° orthotopic IRISOE tumors and were maintained in Dox-supplemented RPMI 1640 medium containing 10% fetal bovine serum (FBS). Human bone marrow-derived MSCs were isolated from volunteers, verified, and propagated by Texas A&M (HSC COM Institute for Regenerative Medicine). Mouse MSCs were obtained from ATCC. In our laboratory, mouse and human MSCs were maintained in MEM/α-GlutaMAX medium supplemented with 17% FBS. All commercial and in-house cell lines were authenticated by STR profiling and tested for mycoplasma contamination.

### Antibodies and drugs

Mouse monoclonal anti-human IRIS and Rabbit polyclonal anti-mouse Iris antibodies were developed in our laboratory. Rabbit polyclonal anti-IL-6R (sc-13947), anti-EP2 (sc-20675), and goat polyclonal anti-EP4 (16022) were from Santa Cruz Biotech. Goat polyclonal anti-gp130 (AF-228-NA) and mouse monoclonal anti-STAT3 (MAB1799) were from R&D Systems. Rabbit polyclonal anti-JAK2 (07-606) and anti-p-JAK2 (06-255-1) were from Millipore Sigma. Rabbit polyclonal anti-Cyclin D1 (RB-010-P0) and mouse monoclonal anti-CK5 (MA5-17057) were from Thermo-Scientific. Rabbit polyclonal anti-IL-6 (ab6672), anti-γ-Tubulin (ab11321), mouse monoclonal anti-CD105 (ab114052), and Rat monoclonal anti-CD90 (ab3105) were from Abcam Inc. Mouse monoclonal anti-survivin (2802), Rabbit polyclonal anti-p-STAT3^Y705^ (9145), and anti-β-actin (4970) were from Cell Signaling Inc. Mouse monoclonal, anti-CK18 (RGE53): Sc-32329 Santa Cruz.

The STAT3 inhibitor (VII, Cat #573103), PI3’K inhibitor (LY294002, Cat #440202), and the ERK inhibitor (PD98059, Cat #51300) were from Calbiochem and were all used at 10-μM working concentration.

### Proliferation assay

Assays were performed using the Promega MTS kit (G3582) following the manufacturer’s instructions.

### Cytokine array

IRISOE1-5 grown in the absence of doxycycline "Dox" (i.e., naïve HME) and in the presence of Dox (i.e., HME/IRIS, *aka* IRISOE1-5) cells was assessed for differential factors. Briefly, conditioned media (CM) from an equal number of either cell line plated in serum-free medium for 20 h under standard conditions were used to screen cytokine, chemokine, and growth factor antibody arrays (RayBio, Norcross, GA, USA), performed according to the manufacturer’s instructions and as described previously [25]. Routinely, three different preparations of each cell line CM were assessed on antibody arrays. Thus, *n* = 3 for each cell line in the presence or absence of Dox.

### siRNA transfection

Naïve HME, IRIS291, and IRIS293 cells were seeded at a density of 3 × 10^5^ cells/well in a 6-well plate. After 16–18 h, transient transfection of siLuc siRNA, siHIF1α (using two different siRNA), and IRIS siRNA was carried out using Xfect™ Transfection reagent (Clonetech) according to the manufacturer’s instructions. After 48 h, media were changed and cells were exposed to normoxia (20% O_2_) or hypoxia (1% O_2_, using Hypoxia Chamber [STEMCELL technologies, #27310]) for an additional 24 h, when CM were collected for ELISA analysis.

### Conditioned media transfer experiment

Briefly, normoxic or hypoxic (24 h) naïve HME, IRIS291, IRIS292, or IRIS293 CM were directly analyzed for secreted factors, and cells for surface receptor expression. Mammary cell CM (24 h), reconditioned by MSC contact (24 h), was analyzed for secreted factors, and MSC for surface receptor expression. Mammary cell CM (24 h) reconditioned by MSC contact (24 h), then reconditioned by the same mammary cell line contact (24 h), was analyzed for secreted factors and mammary cell surface receptor expression. At all steps, equal numbers of each cell type were seeded to avoid discrepancies due to cell number variations. At various steps in this protocol, specific NeuAb or inhibitor was added as indicated in the “Results” section. Secretion was investigated by ELISA, and surface receptors expression by western blotting.

### Cytokine ELISA

Co-culture CM or mice sera diluted in carbonate coating buffer (pH 9.6) were used to coat 96-well ELISA plates overnight at 4 °C. Plates were then washed three times with PBST (phosphate-buffered saline - 0.05% Tween-20) and blocked with 2% bovine serum albumin for 1 h at room temperature (RT). Plates were then incubated with primary antibody diluted in blocking solution for 2 h at room temperature (RT) followed by HRP-conjugated secondary antibody for 1 h at RT. The reaction was read using Western Lightning Plus-ECL (PerkinElmer, Waltham, MA, USA) as a substrate. Experiments were done in triplicates performed three separate times and shown as mean ± SD.

### Co-culture experiment

Boyden chambers (BD Biosciences) of 8-μm-pore size (for migration) or 0.4-μm-pore size (for secretome) analysis were used. IRISOE cells were layered in the lower chamber with or without neutralizing antibodies and test cells were layered in the transwell inserts. Cells migrated to the lower face of the Boyden chamber insert were counted and plotted. Co-cultures were performed under normoxic or hypoxic (for 24 h) conditions.

### Western blot

Briefly, protein lysates were prepared from membrane fraction or whole cell extracts by sonication in PBS containing protease and phosphatase inhibitor tablets (Thermo Scientific), according to the manufacturer’s instructions (performed as previously described [25]). Protein concentration was estimated using the Pierce™ BCA protein assay kit (Thermo Scientific). Cell lysates were denatured in NuPAGE LDS sample buffer (Thermo Scientific) and were resolved on NuPAGE gels (Thermo Scientific) and electro-transferred to PVDF membrane. The membrane was blocked with 5% dry milk for 1 h, washed five times (10 min/each) with PBST and subsequently incubated with primary antibody overnight at 4 °C. The next day, blots were washed five times (10 min/each) with PBST and incubated with HRP-conjugated secondary antibody for 1 h at RT, then washed and developed using Western Lightning Plus-ECL as a substrate. Tubulin and actin were used as an internal loading control.

### Immunohistochemistry

Immunohistochemical analysis was performed as previously described [25] on 5-μm-thick paraffin-embedded sections of tumor tissue excised from IRISOE orthotopic mammary tumor generated in Nu/Nu mice. Briefly, sections were deparaffinized, rehydrated, and washed in PBS. Antigen retrieval for IRIS staining was performed by incubating the slides in pepsin (10 μM) for 20 min at 37 °C. Antigen retrieval for all other antigens was performed by boiling the slides in citrate buffer (pH 6.0) for 10 min in the microwave. Slides were then cooled to RT and washed three times with PBS for 15 min each. Slides were incubated in 3% hydrogen peroxide (H_2_O_2_) for 10 min to block endogenous peroxidase activity unless fluorescence analyses were performed. After washing, slides were blocked with 10% normal goat serum for 1 h at RT, washed, and subsequently probed with primary antibodies overnight at 4 °C in a moist chamber. After three PBS washes, slides were incubated with horseradish peroxidase (HRP) or Alexa Fluor (488, 532, or 568)-conjugated secondary antibody for 1 h at RT (depending on the analysis) and were washed with PBS. Slides that were stained with HRP-conjugated secondary antibody were developed with Vector DAB substrate kit (Vector Laboratories) and counterstained with Meyer’s hematoxylin (Thermo Scientific) for 2 min, washed, dehydrated, and mounted with Permount (Thermo Fisher Scientific). Alternatively, slides that were stained with Alexa Fluor-conjugated secondary antibody were counterstained and mounted with VECTASHIELD mounting medium for fluorescence with DAPI (Vector Laboratories) and were imaged under the microscope.

### MSC lineage differentiation

Differentiation of naïve MSCs into different lineages was performed in parallel. In brief, naïve MSCs were grown for 7 days in MSC growth medium (in the same incubator and laboratory by the investigator) and then transferred to the respective differentiation media, containing selected inducers of differentiation [26, 27]. For instance, for the adipogenic differentiation, naïve MSCs were incubated with dexamethasone, 3-isobutyl-1-methylxanthine (IBMX), insulin, and indomethacin for an additional 7 days with medium changed with factors every third day. For the osteogenic differentiation, naïve MSCs were incubated with dexamethasone, β-glycerphosphate, and ascorbic acid for 3 weeks with medium changed with factors three times a week. For the chondrogenic differentiation, naïve MSCs were cultured in rBMP-6, rTGF-β1, ascorbate-2-phosphate, dexamethasone, and sodium pyruvate-containing medium for 3 weeks and the medium replaced with factors three times a week. Finally, for the fibrogenic differentiation, naïve MSCs were incubated for 2 weeks in CTGF and ascorbic acid, and the medium changed three times a week.

### MSC lineage staining assay

Differentiated MSCs were washed with PBS and fixed in 10% formalin for 1 h at RT and stained for adipogenic fate by incubation for 10 min with Oil-Red O (Sigma) to stain intracellular lipid droplets, for osteogenic fate by incubation with Alizarin Red S (Sigma), for chondrogenic fate by incubation with Alcian blue (Sigma), and finally for fibrogenic fate by incubation with PicroSirius/Direct red 80 (Sigma). Cells were washed with PBS and were photographed under the light microscope. Experiments that were done in triplicates performed three separate times.

### Orthotopic and syngeneic mammary models

All animal experiments were approved by the “Institutional Animal Care and Use Committee” (IACUC) of the University of Mississippi Medical Center and in accordance with the NIH guidelines. Athymic or BALB/c (6–8 weeks old) female mice (numbers are indicated in the “Results” section) were injected in 2° left thoracic mammary fat pad with orthotopic 1° IRISOE mammary tumor cell lines; IRIS291 or IRIS293, or the human TNBC cell lines; MDA-MB-231 or MDA-MB-468 cell lines expressing shCtrl or shIRIS, or the mouse TNBC cell line; 4 T1 expressing shCtrl or shIris1 or shIris2, admixed or not with human or mouse MSCs (at 10:1 ratio). After IRISOE tumors reached a tumor volume indicated in figures and text, mice were randomized, divided into groups that were intratumorally injected with vehicle or IRIS-pep four times every third day for 2 weeks. Animals were monitored for tumor formation or death for indicated times. Tumors were measured every third day by digital caliper, and tumor volume was measured according to the formula: volume = (length × width^2^)/2. At the end of the experiment, mice were sacrificed, and tumor and peripheral blood collected. Excised tumors were divided into several portions. One was flash-frozen and used to generate DNAs, RNAs, and proteins. Another was directly processed to prepare single cells using common protocols [28] to perform FACS analysis for IL-6R and CD90 (or CD29 for the syngeneic model), or to generate 3° orthotopic IRISOE mammary tumors. A third portion was paraffin embedded, sectioned (at 5 μm), and processed for IHC staining using, for instance, CK5, CD90 (or CD29 in the syngeneic model), and IL-6R as described above.

### Fluorescence-activated cell sorting

Single cell suspensions from the in vivo treated tumors were processed for FACS analysis. One million cells were stained with specific antibodies on ice for 1 h. Cells were then washed three times with FACS buffer (1% BSA in PBS) by centrifugation at 2000 rpm at 4 °C for 10 min and further incubated with FITC-conjugated secondary antibody for 30 min in ice. Cells were washed and then analyzed for surface staining on Gallios Flow Cytometer (Beckman Coulter, Pasadena, CA, USA), and data were analyzed using Kaluza Flow Cytometry Analysis Software v 1.2.

### TCGA meta-analysis using Kaplan-Meier plotter

Meta-analysis-based biomarker assessment using the online tool Kaplan-Meier plotter (http://kmplot.com) was used to delineate the association between gene expression of IL-6, PTGER2, and PTGER4 separately or combined with overall survival (OS), distant metastasis-free survival (DMFS), and recurrent-free survival (RFS) of the breast cancer patient’s cohorts. Within each cohort, high expressers and low expressers were analyzed and compared for their OS, DMSF, and RFS, respectively.

### Statistical analysis

Statistical analysis was performed using unpaired, two-tailed Student’s *t* test. In all figures, data represents the mean from at least three separate biological repeats done in at least triplicates each +/−SD, *p < 0.05, **p < 0.01, and ***p < 0.001.

## Results

Immobilized antibody arrays were used to compare the condition media (CM) of normal human mammary epithelial (hereafter HME) and IRISOE (HME cells engineered to express IRIS allele) cells. This analysis showed that compared to HME cells, IRISOE cells CM contained ~ 12-fold higher IL-3, ~ 30% higher IL-4, and ~ 3-fold higher IL-6 but no IL-2, IL-5, or IL-7 in both CM (Additional file 1: Figure S1). IL-3 is a multipotent hematopoietic growth factor that signals through high-affinity receptors composed of a unique alpha subunit and a beta subunit common to granulocyte-macrophage colony-stimulating factor (GM-CSF) and IL-5 [29]. IL-3 induces proliferation, maturation, and probably self-renewal of pluripotent hematopoietic stem cells, in vitro [29]. More importantly, IL-3 supports vessel formation and tumor angiogenesis, in vivo [30]. Interestingly, we recently showed a positive role for IRISOE TNBC cells CM in endothelial progenitor proliferation [12]. Additionally, IL-4 level is high in breast cancer patients who died from ER^−^/PR^−^ tumors [31]. These data suggest IRISOE TNBC tumor cells secrete inflammatory factors with cancer growth and progression promoting roles [32]. We elected to evaluate in detail the role of IL-6 in IRISOE TNBC tumor aggressiveness.

**Fig. 1:**
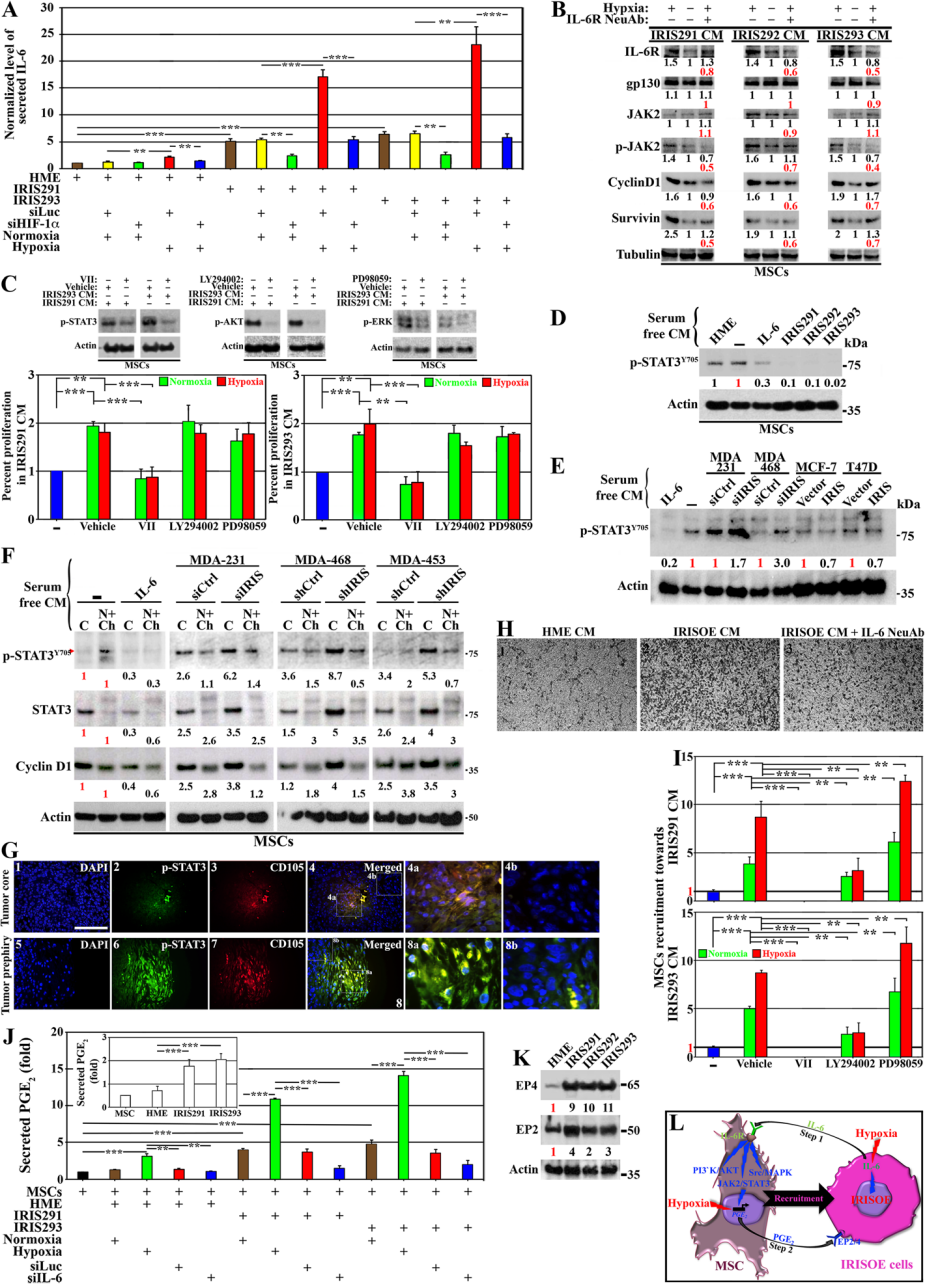
IL-6/PGE_2_ feedback loop between IRISOE TNBC cells and activated MSCs. **a** Normalized IL-6 level detected using ELISA in CM of HME, IRIS291, and IRIS293 cells expressing siLuc or siHIF-1α exposed 24 h to normoxic or hypoxic conditions. Experiments were done three separate times in triplicates. **b** Expression of indicated proteins in MSCs exposed to CM from IRIS291, IRIS292, or IRIS293 cells grown under normoxic or hypoxic condition added *plus* vehicle or anti-IL-6 NeuAb. Fold change in black compares normoxic and hypoxic conditions, and in red compares hypoxia *plus* vehicle vs. *plus* IL-6 NeuAb conditions. Experiments were done at least twice. **c** Top: effects of 10 μM of STAT3 inhibitor; VII, PI3’K/AKT inhibitor; LY294002 and ERK inhibitor; PD98059 on their cognate kinase. Bottom: MTS assay measuring proliferation of MSCs exposed to CM from normoxic or hypoxic IRIS291 or IRIS293 in the presence of vehicle, VII, LY294002, or PD98059. Experiments were done three separate times in triplicates. The level of p-STAT3^Y705^ in MSCs exposed to vehicle [−] or 30 ng/ml rIL-6 or CM from HME, IRIS291, IRIS292, or IRIS293 cells for 30 min (d), or CM from MDA-231 or MDA-468 expressing si/shCtrl or si/shIRIS or MCF7 or T47D expressing vector or IRIS cDNA for 30 min (**e**). **d**, **e** Experiments were done at least twice. **f** The level of p-STAT3^Y705^, total STAT3 or Cyclin D1 in cytoplasm (C) or nuclear/chromatin (N + Ch) fractions of MSCs exposed to vehicle [−] or 30 ng/ml rIL-6 or CM from MDA-231, MDA-468, or MDA-453 expressing si/shCtrl or si/shIRIS for 30 min. Experiments were done at least twice. **g** Fluorescence IHC for p-STAT3^Y705^ and CD105 on sections from the core (1–4) or periphery (5–8) of IRIS291 tumors. Scale bar = 100 μm. **h** Migration in Boyden chambers of MSCs layered in inserts and exposed to HME (1) or IRISOE cells CM *plus* vehicle (2) or *plus* IL-6 NeuAb (3). Experiments were done in triplicates three separate times. **i** Migration in Boyden chambers of MSCs layered in the inserts exposed to vehicle [−], IRIS291 or IRIS293 cell CM containing vehicle, VII, LY294002, or PD98059 (at 10 μM each). Experiments were done three separate times in triplicates. **j** Inset: normalized PGE_2_ level secreted from naïve MSCs, HME, IRIS291, or IRIS293 cells as detected using ELISA. Experiments were done three separate times in triplicates. Normalized PGE_2_ levels secreted from naïve MSCs or MSCs exposed to CM from HME, IRIS291, or IRIS293 cells expressing (48 h) siLuc or siIL-6 and exposed (24 h) to normoxia or hypoxia as detected using ELISA. Experiments were done three separate times in triplicates. **k** The level of EP2 and EP4 in HME, IRIS291, IRIS292, or IRIS293 cells. Experiments were done at least twice. **l** Schematic representation of the data represented above

To accomplish that evaluation, ELISA analysis was performed on CM from normal HME cells, or cells from the 1° orthotopic mammary tumor cell lines; IRIS291 and IRIS293 (see detail analysis for generation of these cell lines above and in [14]). Compared to HME cells, IRIS291 and IRIS293 cells express > 3-fold higher IRIS (Additional file 2: Figure S2A) and show aggressive breast cancer cells morphology (compare Additional file 2: Figure S2F-H to S2E). Accordingly, compared to HME cells, IRIS291 and IRIS293 cells secrete 5–6-fold higher IL-6 (Fig. 1a, brown bars). Exposing these cells to hypoxia (24 h) exacerbated IL-6 secretion to 15–20-fold in IRIS291 and IRIS293 cells (and a slight increase from HME cells, Fig. 1a, red bars). Under normoxic condition, transfection of control siRNA (siLuc) had no effect on IL-6 secretion from HME, IRIS291, or IRIS293 cells (Fig. 1a, yellow bars). In contrast, HIF-1α silencing (using two different siRNAs, see knockdown effect on the level of HIF-1α mRNA and protein in Additional file 3: Figure S3A and B, respectively) blocked IL-6 production/secretion from IRISOE cells under normoxic (Fig. 1a, green bars), as well as under hypoxic condition (Fig. 1a, blue bars). We recently showed that HIF-1α expression is dependent on IRIS expression under normoxic conditions [12]. Taken together, this suggests that under normal conditions, IRISOE elevates HIF-1α expression in TNBC cells leading to IL-6 production/secretion, while hypoxia additively promotes HIF-1α expression/stabilization exacerbating IL-6 production/secretion from TNBC cells.

Next, we evaluated the effect of IL-6 on naïve MSCs (Additional file 2: Figure S2I). We showed that compared to normoxic CM, hypoxic IRIS291, IRIS292, or IRIS293 CM enhanced IL-6R expression, had no effect on gp130 (a co-receptor for IL-6 [33]) or JAK2 (a downstream target of IL-6R [33]) expression, while enhancing the level of phosphorylated/activated JAK2 leading to enhanced Cyclin D1 and survivin (p-JAK2 downstream targets) expression in naïve MSCs (compare 1st to 2nd lanes, Fig. 1b). These effects were blocked when hypoxic IRIS291, 292, and 293 CM were added to naïve MSCs in the presence of IL-6 neutralizing antibody (NeuAb, compare 3rd to 1st, Fig. 1b). Together, these effects suggest that IL-6-rich IRISOE cells CM upregulates IL-6R in neighboring naïve MSCs that complex with the constitutively expressed co-receptor gp130, culminating in promotion of proliferation and survival. Effects are exacerbated under hypoxic conditions, such as those within IRISOE TNBC tumor aggressiveness niche [34].

IL-6/IL-6R-gp130 activates, directly or indirectly signaling from JAK2/STAT3, PI3’K/AKT or c-Src/ERK [35]. To evaluate the contribution of each signaling pathway in the proposed IRISOE TNBC effect on MSC proliferation, we exposed naïve MSCs to IRIS291 CM or IRIS293 CM containing vehicle, 10 μM VII (STAT3 inhibitor), 10 μM LY294002 (PI3’K/AKT inhibitor), or 10 μM PD98059 (MAPK/ERK inhibitor) for 24 h. All drugs significantly reduced the activities of their cognate target (top, Fig. 1c). MTS proliferation assay showed that compared to no-CM (see [−], Fig. 1c), the CM from either cell line triggered ~ 2-fold increase in naïve MSC proliferation in the presence of vehicle (Fig. 1c), reduced by ~ 50% in the presence of VII but not LY294002 or PD98059 (Fig. 1c). Taken together, these responses suggest that JAK2/STAT3 signaling is the primary pathway used by IL-6 secreted from IRISOE TNBC cells to influence MSC proliferation (and perhaps survival).

Therefore, we elected to further pursue the STAT3 pathway. Recently, we generated several doxycycline-inducible IRIS-overexpressing HME cell clones, named IRIS3, 4, 5, 9, 10, 16, and 17 that express > 2-fold higher IRIS (Additional file 4: Figure S4A). Naïve MSCs were exposed 24 h to none, 30 ng/ml recombinant (r)IL-6, and HME cells CM or CM from these clones. Unexpectedly, we found that compared to vehicle, rIL-6 decreased instead of increased the level of p-STAT^Y705^ in naïve MSCs by ~ 30% (Additional file 4: Figure S4B). Moreover, compared to normal HME cell CM, the IL-6-enriched IRISOE TNBC cell CM also reduced the p-STAT3^Y705^ level in naïve MSCs to various degrees (Additional file 4: Figure S4B), suggesting that prolonged exposure to IL-6 suppresses instead of enhances p-STAT3^Y705^. Therefore, we repeated the assay for 30 minutes (30 min) exposure instead. Again, instead of enhancing, rIL-6 suppressed p-STAT3^Y705^ to an even lower level (Additional file 4: Figure S4C). Similarly, compared to HME cell CM, the IL-6-enriched IRISOE cell CM significantly decreased the p-STAT3^Y705^ level in naïve MSCs (Additional file 4: Figure S4C).

We reasoned that IL-6 could interact with soluble IL-6R and/or gp130 (sIL-6R or sgp130) in the serum, which could affect the interaction with receptors on the surface of naïve MSCs. Therefore, we exposed naïve MSCs 30 min to none, 30 ng/ml rIL-6, HME cell CM, and CM from IRIS291, IRIS292, or IRIS293 cells grown (24 h) in serum-free (SF) conditions. Again, naïve MSCs exposed to none or to HME CM showed high-level p-STAT3^Y705^, whereas those exposed to rIL-6 showed 70% decrease, and those exposed to IL-6-enriched IRIS291, IRIS292, or IRIS293 cell CM showed ≥ 90% decrease in the level of p-STAT3^Y705^. To confirm this further, we silenced IRIS in the endogenously overexpressing TNBC cell lines; MDA-231, MDA-468, and MDA-453 using siRNA (target-specific area in IRIS-intron 11, Additional file 2: Figure S2B) or shRNA (targets a different part in IRIS-intron 11, Additional file 2: Figure S2C). We also overexpressed IRIS in the endogenously low expressing lines of luminal cells; MCF7 or T47D (Additional file 2: Figure S2D). These cell lines were grown in SF-media for 24 h, and CM collected were added to naïve MSCs for 30 min. Again, compared to vehicle, rIL-6 reduced the p-STAT3^Y705^ level in naïve MSCs by ~ 80% (Fig. 1e). More importantly, compared to siCtrl-transfected MDA-231 cells CM, siIRIS-transfected MDA-231 cells CM triggered ~ 1.7-fold higher p-STAT3^Y705^ in naïve MSCs, and compared to siCtrl-transfected MDA-468 cells CM, siIRIS-transfected MDA-468 cells CM triggered ~ 3-fold higher p-STAT3^Y705^ in naïve MSCs (Fig. 1e). In contrast, compared to vehicle-expressing MCF-7 or T47D cells CM, IRIS-expressing MCF-7 or T47D cells CM decreased the p-STAT3^Y705^ level by ~ 30% in naïve MSCs (Fig. 1e).

Since phosphorylated STAT3 can be cytoplasmic (C) or nuclear, either soluble (N) or on the chromatin (Ch), we repeated the previous experiment using C and N + Ch extracts instead of total proteins prepared using cell sonication. Again, compared to untreated naïve MSCs, 30 ng/ml rIL-6 treatment suppressed the level of p-STAT3^Y705^ in C and N + Ch by ~ 70%, total STAT3 in C and N + Ch by ~ 70% and ~ 40%, respectively, and the downstream target, Cyclin D1 in C and N + Ch by ~ 60% and ~ 40%, respectively (Fig. 1f). Compared to control cell CM, exposure to IRIS-silenced MDA-231, MDA-468, or MDA-453 cell CM significantly increased the levels of p-STAT3^Y705^, total STAT3, and Cyclin D1 in the cytoplasm of naïve MSCs (Fig. 1f), while decreasing the levels of these proteins in N + Ch fraction of naïve MSCs (Fig. 1f). This suggests that CM from IRISOE TNBC cells enhances p-STAT3^Y705^ accumulation in the cytoplasm, restricts its nuclear translocation, and/or enhances p-STAT3^Y705^ dephosphorylation in the nucleus and/or the cytoplasm.

To further illustrate this, we immunohistochemically (IHC) stained sections from the 1° IRISOE TNBC orthotopic mammary tumor (see above and [12]) with p-STAT3^Y705^ and the MSC marker, CD105 [36] antibodies. p-STAT3^Y705^-positive cells were present within the core (Fig. 1g_2_) as well as the periphery (Fig. 1g_6_) of the tumor. The majority of these cells were also CD105^+^ (Fig. 1g_3 and 7_), suggesting that the majority of the activated p-STAT3^Y705^ within IRISOE TNBC tumors exists in MSCs, not tumor cells. In fact, higher magnification images clearly showed that the majority of p-STAT3^Y705^-positive cells are also CD105^+^ (Fig. 1h_4a and 8a_), and p-STAT3^Y705^-negative are CD105^−^ (Fig. 1g_4b and 8b_). We also noticed that the staining for both “p-STAT3^Y705^ and CD105” were much more intense at the periphery than the core of the tumor (compare Fig. 1g_4a_ to g_8a_). These observations suggest that within IRISOE TNBC tumors, p-STAT3^Y705^ is confined to MSCs. To clarify that these CD105^+^ cells are indeed MSCs recruited from mouse bone marrow and not, although highly unlikely, some IRISOE cells that upregulated CD105 expression, we co-stained adjacent sections with mouse-specific CD90 antibody and the CD105 antibody. This co-staining unequivocally proved that these are indeed mouse MSCs (Additional file 5: Figure S5).

In inserts of Boyden chambers, we layered naïve MSCs, and in the bottom wells, we added HME or IRISOE TNBC cells CM in the absence or presence of IL-6 NeuAb for 48 h. Medium + Ab was changed daily. Compared to CM from HME cells, IRISOE cell CM attracted more MSCs to the lower side of the inserts in the absence (compare 2nd to 1st, Fig. 1h) not the presence (compare 3rd to 2nd, Fig. 1h) of IL-6 NeuAb. To clarify which signaling pathway downstream of the IL-6/IL-6R-gp130 is involved in this recruitment, we repeated the Boyden chamber assay in the presence of VII, LY294002, or PD98059 (same concentrations as above). Compared to the no-CM treatment, IRIS291 or IRIS293 CM promoted 5–10-fold increase in naïve MSC recruitment to the lower side of the inserts in the presence of vehicle (Fig. 1i). This recruitment was completely blocked in the presence of VII (Fig. 1i), partially blocked in the presence of LY924002 (Fig. 1i), while enhanced instead in the presence of PD98059 (Fig. 1i). Results suggest that STAT3 signaling promotes MSC proliferation and migration, AKT signaling promotes migration not proliferation, and ERK signaling had no effect on proliferation while inhibiting MSC migration.

Within tumors, MSCs are a source of pro-inflammatory cytokines, such as PGE_2_ [37–40], that promote migration of endothelial cells [40] and macrophages [12] into tumors. However, the effect of MSCs on tumor cells is less defined. To study that in our model, CM from IRIS291 or IRIS293 was first analyzed by ELISA for the level of PGE_2_. Each cell line secretes ~ 2-fold higher PGE_2_ compared to HME or naïve MSCs (Fig. 1j, inset). Moreover, compared to unexposed, or exposed to HME cells CM, naïve MSCs exposed to normoxic IRIS291 or IRIS293 cell CM secrete > 5-fold higher PGE_2_ (Fig. 1j, black and brown bar, respectively). The secretion increased to > 10-fold when naïve MSCs were instead exposed to hypoxic (24 h) IRIS291 CM or hypoxic IRIS293 CM (green bars, Fig. 1j). Even those exposed to CM from hypoxic HME cells secreted higher PGE_2_ (green bars, Fig. 1j). More importantly, compared to control cell CM grown under normoxia, CM from HME, IRIS291, or IRIS293 cells silenced from IL-6 did not induce PGE_2_ secretion from naïve MSCs (compare blue to red bars, Fig. 1j). Taken together, observations indicate that IL-6 secreted from IRISOE TNBC cells triggers MSCs to secrete PGE_2_.

Finally, we found that compared to HME cells, IRIS291, IRIS292, and IRIS293 cells express much higher levels of the PGE_2_ receptors: EP2 and EP4 (Fig. 1k, two of the most abundant PGE_2_ receptors in TNBCs [41, 42]). Our findings suggest that high-level IL-6 secreted by IRISOE TNBC cells (especially those exposed to hypoxia within the aggressiveness niche [34]) triggers naïve MSCs to express IL-6R, which activates STAT3, AKT, and ERK signaling in them (step 1, Fig. 1l). These signaling pathways affect the proliferation and/or migration ability of MSCs; for instance, into IRISOE TNBC tumors. IL-6 also triggers naïve MSCs to secrete PGE_2_ that reciprocally activates IRISOE expressing higher levels of the receptors EP2 and EP4 (step 2, Fig. 1l).

### In vivo evidence for the IL-6/PGE_2_-positive feedback loop

**Fig. 2:**
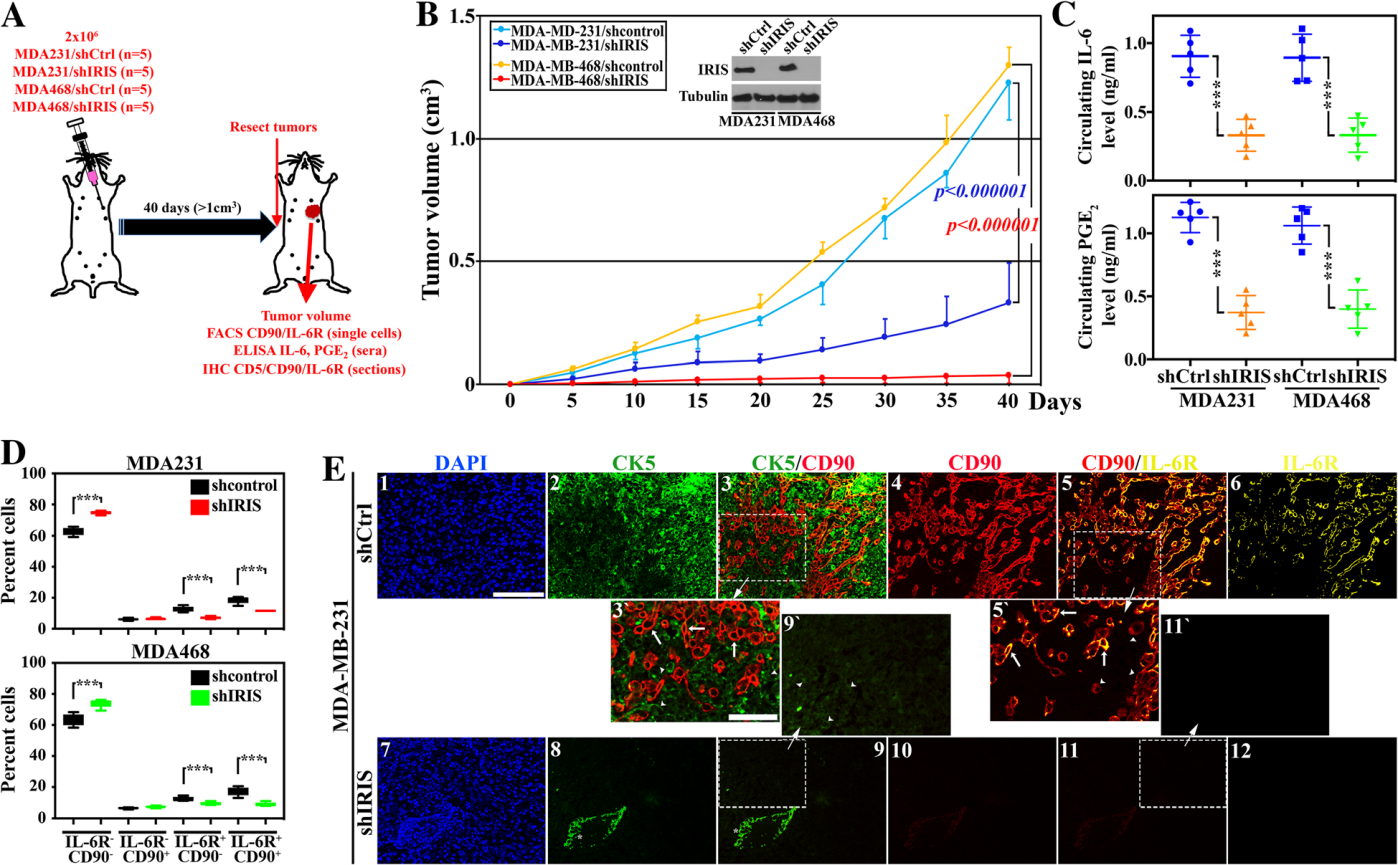
IL-6-secreting TNBC cells attract IL-6R-expressing/PGE_2_-secreting MSCs, in the orthotopic model. **a** Schematic representation of the assays performed. **b** The volume of orthotopic mammary tumors developed following injection of 2 × 10^6^ cells of MDA-231/shCtrl cells (light blue, *n* = 5), MDA-231/shIRIS (dark blue, *n* = 5), MDA-468/shCtrl (yellow, *n* = 5), and MDA-468/shIRIS (red, *n* = 5). The inset shows the expression of IRIS in these cells. **c** The levels of circulating IL-6 (upper) or PGE_2_ (lower) as detected using ELISA in sera from mice bearing MDA-231/shCtrl or MDA-231/shIRIS and MDA-468/shCtrl, or MDA-468/shIRIS tumors. **d** FACS analysis of the percentage of IL-6R^−^CD90^−^, IL-6R^−^CD90^+^, IL-6R^+^CD90^−^, or IL-6R^−^CD90^+^ cells within MDA-231/shCtrl and MDA-231/shIRIS tumors (upper), or MDA-468/shCtrl and MDA-468/shIRIS tumors (lower). Fluorescence IHC staining of MDA-231/shCtrl or MDA-231/shIRIS tumor sections with CK5 (green), the MSC marker CD90 (red), and IL-6R (yellow). In 3`, 5`, and 9`, arrowheads denote cancer cells and arrows denote MSCs. The asterisk in 8 denotes immune cells (e.g., B cells) or red blood cells non-specific labeling with most likely a subset of secondary antibodies. Scale bars = 100 μm in 1–6 and 7–12 and 50 μm in 3`, 9`, 5`, and 11`

To expand the data in vivo, 20 athymic mice were injected with MDA-231/shCtrl (*n* = 5), MDA-231/shIRIS (*n* = 5), MDA-468/shCtrl (*n* = 5), and MDA-468/shIRIS (*n* = 5) in the 2nd left thoracic mammary fat pads. At ~ 1.0 cm^3^ or at 40 days, tumors were resected, and peripheral bloods were collected from all mice (Fig. 2a). Consistent with our previously published results [15], TNBC cells lacking IRIS expression (Fig. 2b, inset) had a significantly reduced ability to develop tumors. Indeed, tumors formed using MDA-231/shIRIS cells were ~ 30% the volume of tumors formed using MDA-231/shCtrl cells (compare dark to light blue lines, Fig. 2b), and tumors formed using MDA-468/shIRIS cells were ~ 3% the volume of tumors formed using MDA-468/shCtrl cells (compare red to yellow lines, Fig. 2b).

Moreover, according to ELISA analysis, sera isolated from peripheral blood (PB) from naïve mice (*n* = 20) contained ~ 0.001 ng/ml IL-6. In comparison, ~ 900-fold higher IL-6 was measured in sera from MDA-231/shCtrl tumor-bearing mice (0.91 ± 0.15 ng/ml, Fig. 2c, upper left blue) or MDA-468/shCtrl tumor-bearing mice (0.89 ± 0.17 ng/ml, upper right blue). In contrast, sera from MDA-231/shIRIS tumor-bearing mice contained 0.33 ± 0.11 ng/ml, an ~ 65% decrease in the level of circulating IL-6 in these mice (*p = 0.0001*, Fig. 2c, upper left orange), and sera from MDA-468/shIRIS tumors-bearing mice contained 0.30 ± 0.12 ng/ml, an ~ 66% decrease in the level of circulating IL-6 in these mice (*p = 0.0003*, Fig. 2c, upper right green).

Furthermore, sera from naïve mice (*n* = 20) contained ~ 0.003 ng/ml PGE_2_ (not shown). In comparison, > 350-fold higher PGE_2_ was measured in sera from MDA-231/shCtrl tumor-bearing mice (1.12 ± 0.12 ng/ml, lower left blue) or MDA-468/shCtrl tumor-bearing mice (1.1 ± 0.15 ng/ml, lower right blue). In contrast, sera from MDA-231/shIRIS tumor-bearing mice contained 0.37 ± 0.13 ng/ml, an ~ 67% decrease in the level of circulating PGE_2_ in these mice (*p = 0.000001*, Fig. 2c, lower left orange), and MDA-468/shIRIS tumor-bearing mice contained 0.39 ± 0.15 ng/ml, an ~ 65% decrease in the level of circulating PGE_2_ in these mice (*p = 0.0001*, Fig. 2c, lower right green).

Additionally, a fresh portion of each tumor was used to generate single cells and immediately labeled with anti-IL-6R and -CD90 antibodies. FACS analysis of these cells showed that compared to MDA-231/shCtrl tumors, MDA-231/shIRIS tumors contained more IL-6R^−^/CD90^−^ (i.e., double negative [DN], 62.8 ± 2.5% vs. 74.9 ± 1.1, *p < 0.0000001*, Fig. 2d, upper), unchanged numbers of IL-6R^−^/CD90^+^ (i.e., CD90^+^, 6.1 ± 0.7% vs. 6.4 ± 0.7, *p = 0.5*, Fig. 2d, upper), and decreased numbers of IL-6R^+^/CD90^−^ (i.e., IL-6R^+^, 12.6 ± 1.7% vs. 7.1 ± 0.9, *p = 0.0002*, Fig. 2d, upper) and IL-6R^+^/CD90^+^ (i.e., double positive [DP], 18.6 ± 2.2% vs. 11.6 ± 0.8, *p = 0.0002*, Fig. 2d, upper) cells. Similarly, compared to MDA-468/shCtrl tumors, MDA-468/shIRIS tumors also contained more DN (63.4 ± 3.8% vs. 74.1 ± 2.7, *p = 0.001*, Fig. 2d, lower), unchanged numbers of CD90^+^ (6.6 ± 0.5% vs. 7.3 ± 0.7, *p = 0.9*, Fig. 2d, lower), and decreased numbers of IL-6R^+^ (12.5 ± 1.3% vs. 9.6 ± 1.1, *p = 0.004*, Fig. 2d, lower) and DP (17.5 ± 2.8% vs. 9.1 ± 1.2, *p = 0.0003*, Fig. 2d, lower) cells.

Finally, for further confirmation, a paraffin-embedded portion of each tumor was cut at 5 μm and IHC stained using anti-CK5 (basal marker), anti-CD90 (infiltrated mouse MSC-specific marker [12]), and anti-IL-6R antibodies. As expected, in MDA-231/shCtrl tumors (Fig. 2e_1_), almost all the cells were CK5^+^ (Fig. 2e_2 and 3` arrowheads_). There were also high numbers of CD90^+^ cells (Fig. 2e_4 and 5` arrowheads_) and IL-6R^+^ cells (Fig. 2e_6_). However, while none of the CD90^+^ cells were CK5^+^ cells (compare arrowheads and arrows, respectively in Fig. 2e_3`_), the majority were IL-6R^+^ cells (compare arrowheads and arrows, respectively Fig. 2e_5`_). In contrast, in the MDA-231/shIRIS tumor section, there was almost a complete absence of the high CK5 expression in the majority of cells (note very low-level expression in Fig. 2e_8 and 9` arrowheads_). There was a complete absence of CD90^+^ cells (Fig. 2e_10 and 11` arrowheads_) and IL-6R^+^ cells (Fig. 2e_12_) within these tumors. To define the nature of the CK5^−^ cells in IRIS-silenced MD-A231 tumors, we stained sections from these tumors for the luminal cytokeratin biomarker CK18. As expected, while no/very low CK18 staining was detected in MDA-231/shCtrl tumors, high C18 staining was detected in MDA-231/shIRIS tumors (compare A and B in Additional file 6: Figure S6). In fact, IRIS-silencing converted the aggressive morphology of the TNBC cells—MDA-231, MDA-468, and MDA-453—into the less aggressive morphology of luminal A cells (compare K, M, and O to J, L, and N, respectively, Additional file 2: Figure S2). Moreover, IRISOE converted the less aggressive morphology of the luminal A cells, MCF-7 and T47D, into aggressive TNBC cell morphology (compare Q and S to P and R, respectively, Additional file 2: Figure S2). The results suggest that IL-6R is expressed primarily by the infiltrated MSCs—not IRISOE TNBC tumor cells—and that even in vivo, suppressing IRIS expression in IRISOE TNBC cells converts them into luminal A tumor cells, correlated with suppression in IL-6 secretion, and prevention in CD90^+^/IL-6R^+^ MSC recruitment into IRISOE tumors; especially the aggressiveness niche [12, 34] and their conversion into PGE_2_-secreting cells.

### Further in vivo evidence for the IL-6/PGE_2_-positive feedback loop

**Fig. 3:**
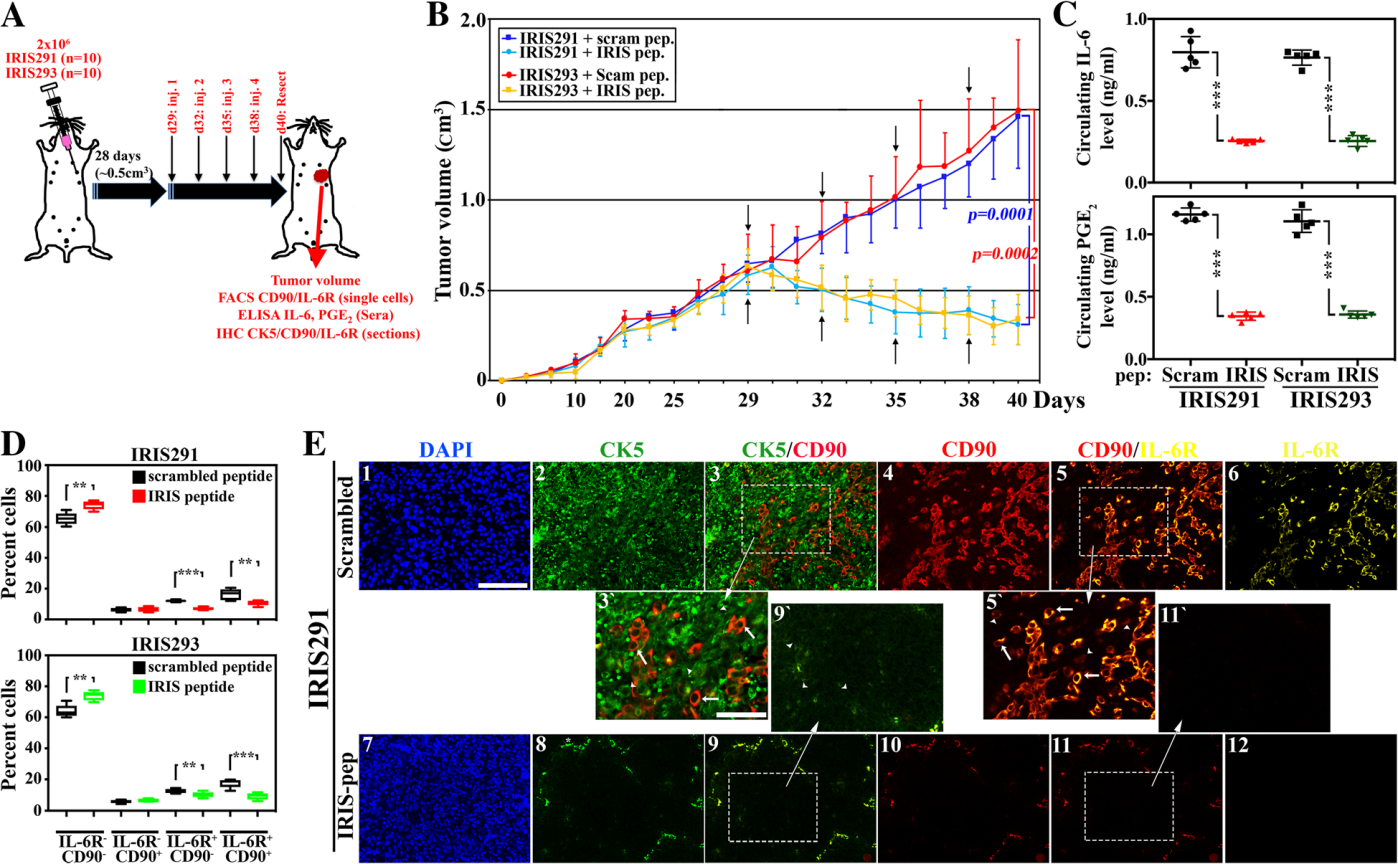
IRIS inactivation suppresses recruitment of the IL-6R-expressing/PGE_2_-secreting MSCs in an established TNBC orthotopic model. **a** Schematic representation of the assays performed. **b** The volume of orthotopic mammary tumors developed following injection of 2 × 10^6^ IRIS291 cells and at ~ 0.5 cm^3^ intratumorally (i.t.) injected with scrambled (dark blue, *n* = 5) or IRIS-pep (light blue, *n* = 5), or 2 × 10^6^ IRIS293 cells and at 0.5 cm^3^ i.t. injected with scrambled (red, *n* = 5) or IRIS-pep (yellow, *n* = 5). **c** The levels of circulating IL-6 (upper) or PGE_2_ (lower) as detected using ELISA in mice bearing IRIS291 or IRIS293 tumors treated with scrambled or IRIS-pep. **d** FACS analysis of the percentage of IL-6R^−^CD90^−^, IL-6R^−^CD90^+^, IL-6R^+^CD90^−^, or IL-6R^−^CD90^+^ cells within IRIS291 treated with scrambled or IRIS-pep (upper), as well as IRIS293 treated with scrambled or IRIS-pep (lower). **e** Fluorescence IHC staining with CK5 (green), the MSC marker CD90 (red), and IL-6R (yellow) of the 5-μm-thick section from IRIS291 tumors treated with scrambled or IRIS-pep as per scheme shown in **a**. In 3`, 5`, and 9`, arrowheads denote cancer cells and arrows denote MSCs. The asterisk in 8 denotes immune cells (e.g., B cells) or red blood cells non-specific labeling with most likely a subset of secondary antibodies. Scale bars = 100 μm in 1–6 and 7–12 and 50 μm in 3`, 9`, 5`, and 11`

To analyze the feedback following inhibition of IRIS activity in established tumors in vivo, a second set of athymic mice (*n* = 20) were injected with IRIS291 (*n* = 10) or IRIS293 (*n* = 10) in the 2nd left thoracic mammary fat pads (Fig. 3a). At ~ 0.5 cm^3^, each set was randomized and intratumorally injected four times, once every 3 days with scrambled peptide (*n* = 5) or the IRIS-inhibitory peptide (IRIS-pep, *n* = 5, Fig. 3a). As we previously reported, IRIS-pep treatment leads to loss of IRIS protein expression [15, 25]. Moreover, while IRISOE TNBC tumors injected with scrambled peptide continued to grow to reach a volume of > 200% within this period, the IRIS-pep-treated tumors regressed by ~ 50% (Fig. 3b). Indeed, scrambled peptide-treated IRIS291 tumors were ~ 1.4 cm^3^ at resection, while IRIS-pep-treated IRIS291 tumors regressed to < 0.3 cm^3^ (*p = 0.002*, compare the dark to light blue lines, Fig. 3b) and scrambled peptide-treated IRIS293 tumors were ~ 1.5 cm^3^ at resection, while IRIS-pep-treated IRIS293 tumors regressed to < 0.3 cm^3^ (*p = 0.001*, compare red to yellow lines, Fig. 3b).

Again, sera from PB from naïve mice (*n* = 20) contained ~ 1 pg/ml IL-6 and ~ 3 pg/ml PGE_2_ as detected by specific ELISA (not shown). In comparison, > 750-fold higher IL-6 was measured in sera from IRIS291 tumor-bearing mice injected with scrambled peptide (0.79 ± 0.09 ng/ml, Fig. 3c, upper left black) or IRIS293 tumor-bearing mice injected with scrambled peptide (0.76 ± 0.05 ng/ml, Fig. 3c, upper right black). In contrast, sera from IRIS291 tumor-bearing mice injected with IRIS-pep contained 0.25 ± 0.01 ng/ml, an ~ 69% decrease in the level of circulating IL-6 in these mice (*p < 0.000001*, Fig. 3c, upper left red), and sera from IRIS293 tumor-bearing mice injected with IRIS-pep contained 0.25 ± 0.03 ng/ml, an ~ 67% decrease in the level of circulating IL-6 in these mice (*p = 0.0000001*, Fig. 3c, upper right green). Furthermore, > 350-fold higher PGE_2_ was measured in sera from IRIS291 tumor-bearing mice injected with scrambled peptide (1.16 ± 0.05 ng/ml, Fig. 3c, lower left black) or IRIS293 tumor-bearing mice injected with scrambled peptide (1.1 ± 0.09 ng/ml, Fig. 3c, lower right black). Similarly, sera from IRIS291 tumor-bearing mice injected with IRIS-pep contained 0.34 ± 0.03 ng/ml, an ~ 72% decrease in the level of circulating PGE_2_ in these mice (*p = 0.000001*, Fig. 3c, lower left red), and IRIS293 tumor-bearing mice injected with IRIS-pep contained 0.36 ± 0.03 ng/ml, an ~ 67% decrease in the level of circulating PGE_2_ in these mice (*p = 0.0000001*, Fig. 3c, lower right green).

Furthermore, FACS analysis on dissociated cells from these tumors showed that compared to IRIS291 tumors from mice injected with scrambled peptide, tumors from mice injected with IRIS-pep contained more DN (65.5 ± 3.9% vs. 74.91 ± 2.7, *p < 0.003*, Fig. 3d, upper), unchanged numbers of CD90^+^ (6.2 ± 1.3% vs. 6.6 ± 1.6, *p = 0.7*, Fig. 3d, upper), and decreased numbers of IL-6^+^ (12.1 ± 0.8% vs. 7.0 ± 0.9, *p = 0.000001*, Fig. 3d, upper) and DP (16.2 ± 3.6% vs. 10.9 ± 1.7, *p = 0.02*, Fig. 3d, upper) cells. Similarly, compared to IRIS293 tumors from mice injected with scrambled peptide, tumors from mice injected with IRIS-pep contained more DN (64.1 ± 4.1% vs. 73.9 ± 2.9, *p = 0.002*, Fig. 3d, lower), unchanged numbers of CD90^+^ (5.9 ± 1.0% vs. 6.7 ± 0.9, *p = 0.2*, Fig. 3d, lower), and decreased numbers of IL-6^+^ (12.5 ± 1.4% vs. 10.2 ± 1.7, *p = 0.04*, Fig. 3d, lower) and DP (17.5 ± 2.8% vs. 9.3 ± 2.1, *p = 0.0007*, Fig. 3d, lower) cells.

Once more, IHC analysis of paraffin-embedded sections from these tumors showed that in scrambled peptide-treated IRIS291 tumor sections (Fig. 3e_1_), almost all the cells were CK5^+^ (Fig. 3e_2 and 3` arrowheads_). There were also high numbers of CD90^+^ cells (Fig. 3e_4 and 5` arrowheads_), and IL-6R^+^ cells (Fig. 3e_6_). Again, none of the CD90^+^ cells were CK5^+^ cells (compare arrowheads and arrows, respectively in Fig. 3e_3`_), whereas the majority of the CD90^+^ cells were IL-6R^+^ cells as well (compare arrowheads and arrows, respectively Fig. 3E_5`_). In contrast, in IRIS-pep-treated IRIS291 tumors, there was a significant decrease in the CK5 expression in the cells of these tumors (Fig. 3e_8 and 9` arrowheads_). There was also a total absence of CD90^+^ cells (Fig. 3e_10 and 11` arrowheads_) and IL-6R^+^ cells (Fig. 3e_12_). According to IHC staining, the CK5^−^ cells in IRIS-pep-treated IRIS291 tumors stained positive for the luminal cytokeratin biomarker, CK18 (compare c and d, Additional file 6: Figure S6). Altogether, this suggests that IRISOE drives the TNBC phenotype and the aggressiveness of these tumors through MSC recruitment into tumors. Inactivating IRIS in vivo also converts TNBC tumors into luminal A tumor cells, blocks MSC recruitment into tumors, and significantly suppresses the secretion of high-level IL-6 and PGE_2_ into patients’ circulation.

### The IL-6/PGE_2_-positive feedback loop is also observed in the immune-competent model

**Fig. 4:**
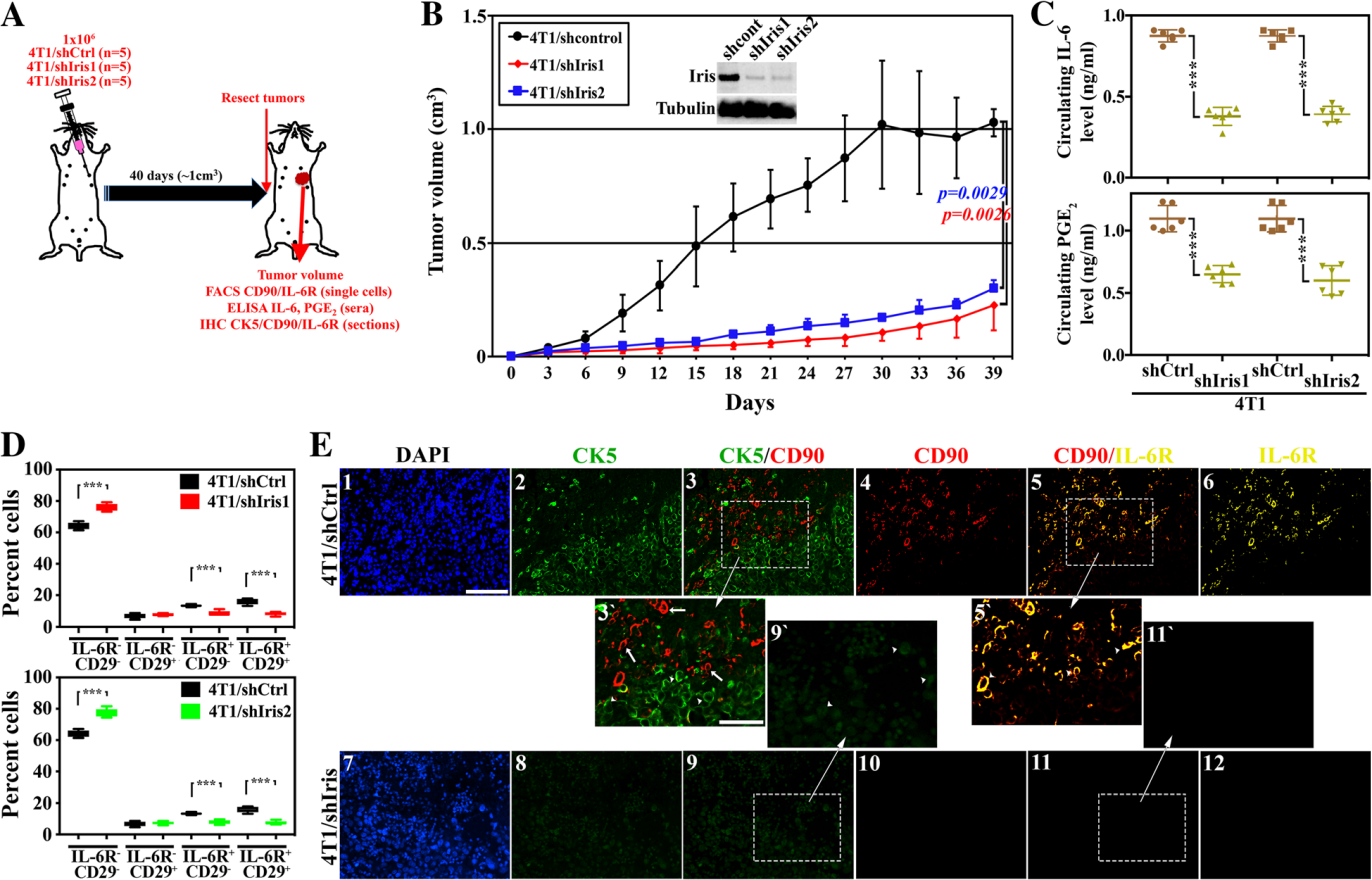
IL-6-secreting TNBC cells attract IL-6R-expressing/PGE_2_-secreting MSCs, in the syngeneic model. **a** Schematic representation of the assays performed. **b** The volume of syngeneic mammary tumors developed following injection of 1 × 10^6^ cells of 4T1/shCtrl (black, *n* = 10), 4T1/shIris1 (red, *n* = 10), or 4T1/shIris2 (blue, *n* = 10) cells. The inset shows the expression of Iris in these cells. **c** The levels of circulating IL-6 (upper) or PGE_2_ (lower) as detected using ELISA in mice bearing 4T1/shCtrl, 4T1/shIris1, or 4T1/shIris2 tumors. **d** FACS analysis of the percentage of IL-6R^−^CD29^−^, IL-6R^−^CD29^+^, IL-6R^+^CD29^−^, or IL-6R^−^CD29^+^ cells within 4T1/shCtrl and 4T1/shIris1 tumors (upper), or 4T1/shCtrl and 4T1/shIris2 tumors (lower). **e** Fluorescence IHC staining of MDA231/shCtrl or MDA231/shIris1 tumor sections with CK5 (green), the MSC marker CD90 (red) and IL-6R (yellow). In 3`, 5`, and 9`, arrowheads denote cancer cells and arrows denote MSCs. Scale bars = 100 μm in 1–6 and 7–12 and 50 μm in 3`, 9`, 5`, and 11`

Similar to human IRIS, mouse Brca1-Iris (hereafter Iris) is overexpressed in mouse breast cancer cell lines, especially those of the TNBC subtype (e.g., 4 T1, generated from a spontaneous BALB/c TNBC tumor [43] and EO771, generated from a spontaneous C57BL/6 TNBC tumor [44]) (not shown). Thus, to analyze the effect of inhibiting Iris expression in establishment of syngeneic TNBC tumors and their phenotype in vivo, we injected 15 BALB/c mice with 4 T1-expressing shCtrl (*n* = 5), -shIris1 (*n* = 5), or -shIris2 (*n* = 5) (two shRNA that target different areas of mouse Iris intron 11 domain [45], Fig. 4a). Tumors developed from 4T1/shCtrl cells grow quickly to reach ~ 1.0 cm^3^ in volume (within 40 days). 4T1/shIris1 (*p = 0.0026*, compare red to black lines, Fig. 4b) or 4T1/shIris2 (*p = 0.0029*, compare blue to black lines, Fig. 4b) tumors grow much slower, and by 40 days, they were ~ 0.2 cm^3^ only.

Here too, ELISA analysis of sera isolated from the PB of these mice showed that the circulating levels of IL-6 in mice bearing 4T1/shIris1 or 4T1/shIris2 tumors were ~ 56% and 55% lower, respectively, compared to the circulating level in mice bearing 4T1/shCtrl tumors (0.39 ± 0.05 and 0.38 ± 0.06 vs. 0.87 ± 0.04, *p < 0.00000001* for both, Fig. 4c, upper). Similarly, the circulating levels of PGE_2_ in mice bearing 4T1/shIris1 or 4T1/shIris2 tumors were ~ 41% and 46% lower, respectively, compared to mice bearing 4T1/shCtrl tumors (0.65 ± 0.07 ng/ml and 0.6 ± 0.12 ng/ml vs. 1.10 ± 0.11 ng/ml, *p = 0.000001* for both, Fig. 4c, lower).

Furthermore, FACS analysis with anti-IL-6R and anti-CD29 (a second mouse MSC-specific marker [12]) antibodies of single cell populations prepared from these tumors showed that compared to 4T1/shCtrl tumors, 4T1/shIris1 tumors contained more DN (64.1 ± 3.9% vs. 75.8 ± 2.2, *p = 0.00004*, Fig. 4d, upper), unchanged numbers of CD29^+^ (6.8 ± 1.5% vs. 7.6 ± 0.9, *p = 0.4*, Fig. 4d, upper), and decreased numbers of IL-6^+^ (13.3 ± 0.8% vs. 8.4 ± 1.6, *p = 0.00002*, Fig. 4d, upper) and DP (16.8 ± 1.9% vs. 8.2 ± 1.1, *p = 0.0002*, Fig. 4d, upper) cells. Similarly, compared to 4T1/shCtrl tumors, 4T1/shIris2 tumors contained more DN (64.1 ± 2.2% vs. 77.4 ± 2.8, *p = 0.00002*, Fig. 4d, lower), unchanged numbers of CD29^+^ (6.8 ± 1.5% vs. 7.3 ± 1.0, *p = 0.5*, Fig. 4d, lower), and decreased numbers of IL-6^+^ (13.3 ± 0.8% vs. 7.8 ± 1.9, *p = 0.00004*, Fig. 4d, lower) and DP (15.8 ± 1.9% vs. 7.5 ± 1.1, *p = 0.0001*, Fig. 4d, lower) cells.

IHC staining of paraffin-embedded sections from these tumors showed that the majority of the cells in 4T1/shCtrl tumors (Fig. 4e_1_) are CK5^+^ cells (Fig. 4e_2 and 3` arrowheads_). There was a large number of CD90^+^ cells (Fig. 4e_4 and 5` arrowheads_) and IL-6R^+^ cells (Fig. 3e_6_) within these tumors as well. Moreover, none of the CD90^+^ cells were CK5^+^ cells (compare arrowheads and arrows, respectively, in Fig. 4e_3`_), while almost all CD90^+^ cells were IL-6R^+^ cells as well (compare arrowheads and arrows, respectively, Fig. 4e_5`_). In contrast, in 4T1/shIris1 tumors (Fig. 4e_7_), we observed a significant reduction in CK5 expression in tumor cells (Fig. 4e_8 and 9` arrowheads_), a complete absence of the CD90^+^ cells (Fig. 4e_10 and 11` arrowheads_), and the IL-6R^+^ cells (Fig. 4e_12_). Identical results were obtained using sections from 4T1/shIris2 tumors (not shown). Moreover, here too, IRIS-silenced tumors showed much higher expression of the luminal biomarker, CK18 (compare f to e, Additional file 6: Figure S6). This again suggests that inhibiting Iris expression can also block recruitment of MSCs into mouse Iris-overexpressing TNBC tumors in immune competent mice leading to loss of tumor aggressiveness.

### IRISOE TNBC cells activate MSC fibrogenic PGE_2_-secreting fate, in vitro

We exposed naïve MSCs to CM from HME, IRIS291, IRIS293, MDA-231/shCtrl, MDA-231/shIRIS, MDA-468/shCtrl, or MDA-468/shIRIS for 7 days (changed daily), followed by staining cells with Oil-Red O (adipogenic marker), Alizarin (osteogenic marker), Alcian blue (chondrogenic marker), or PicroSirius (fibrogenic marker).

**Fig. 5:**
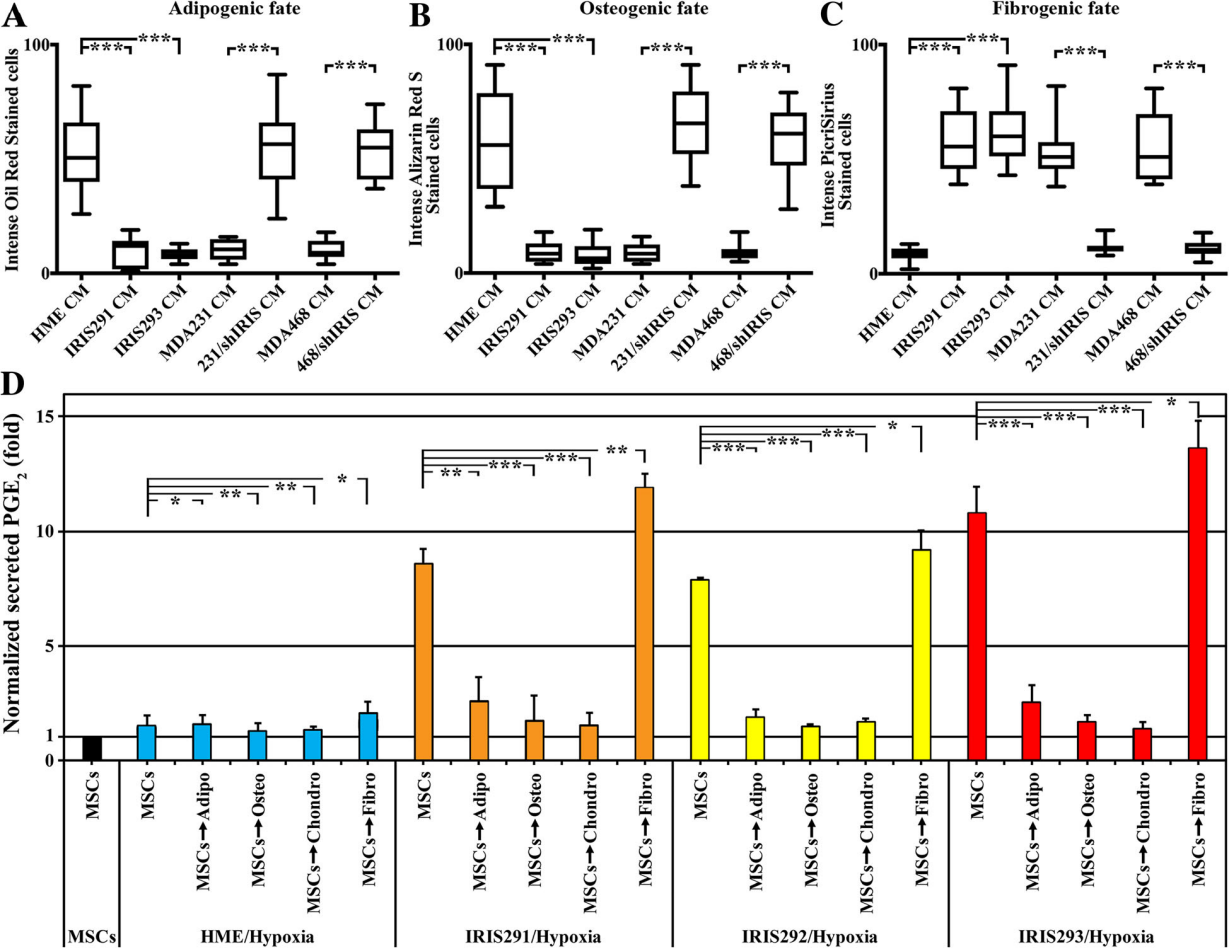
IRISOE TNBC tumor cells skew MSCs toward the PGE_2_-secreting fibrogenic fate, in vitro. Percentage of MSCs stained with Oil Red (**a**), Alizarin Red S (**b**), and PicroSirius (**c**) in cultures exposed to HME, IRIS291, IRIS293 cells, MDA-231/shCtrl, MDA-231/shIRIS, MDA-468/shCtrl, and MDA-468/shIRIS cell CM. **d** The secretion of PGE_2_ from naïve MSCs (black) or MSCs chemically differentiated to adipogenic, osteogenic, chondrogenic, or fibrogenic fates, before they were exposed to hypoxic HME (blue), hypoxic IRIS291 (orange), hypoxic IRIS292 (yellow), or hypoxic IRIS293 (red) cell CM

Naïve MSCs exposed to HME cell CM maintained their naïve MSC morphology of large nuclei and cytoplasm (compare Additional file 2: Figure S2I and Additional file 7: Figure S7A arrow) and equal ability for adipogenic (Fig. 5a and Additional file 7: Figure S7B), osteogenic (Fig. 5b and Additional file 7: Figure S7C), chondrogenic (not shown), and fibrogenic (Fig. 5c and Additional file 7: Figure S7D) differentiation. In contrast, naïve MSCs exposed to CM from IRIS291 and IRIS293 showed more elongating and fibroblastic morphology (compare Additional file 7: Figure S7E and 7I to 7A) and a greater tendency to adopt the fibrogenic fate (Fig. 5a–c and compare Additional file 7: Figure S7H and 7 L to 7D) over the adipogenic (Fig. 5a–c and compare Additional file 7: Figure S7F and S7 J to S7B), the osteogenic (Fig. 5a–c and compare Additional file 7: Figure S7G and S7 K to S7C), or the chondrogenic (not shown) fates.

Significantly, like naïve MSCs exposed to CM from IRISOE tumor cells (see above), those exposed to CM from MDA-231/shCtrl or MDA-468/shCtrl CM also showed more elongating and fibroblastic morphology (compare Additional file 7: Figure S7M and S7 U to S7A) and a greater tendency to adopt the fibrogenic fate (Fig. 5a–c and compare Additional file 7: Figure S7P and S7X to S7D) over the adipogenic (Fig. 5a–c and compare Additional file 7: Figure S7N and S7 V to S7B), the osteogenic (Fig. 5a–c and compare Additional file 7: Figure S7O and S7 W to S7C), or the chondrogenic (not shown) fates. By contrast, naïve MSCs exposed to MDA-231/shIRIS or MDA-468/shIRIS CM maintained morphology resembling naïve MSCs with large nucleus and cytoplasm (compare Additional file 7: Figure S7Q and S7Y to Additional file 2: Figure S2I and Additional file 7: Figure S7A) and showed a greater tendency to adopt all fates equally (Fig. 5a–c and Additional file 7: Figure S7R-T and S7Z-BB, respectively). These data suggest that IRISOE TNBC cell secretome (e.g., IL-6) skews MSC differentiation toward the aggressiveness-promoting CAF fate, in vitro.

If true, we expected that only MSCs induced to adopt the fibrogenic fate would be PGE_2_-secreting cells. To test that, we again chemically induced adipogenic, osteogenic, chondrogenic, or fibrogenic fates in naïve MSCs and then exposed each for 24 h to the CM from normal HME, IRIS291, IRIS292 or IRIS293 cells that were exposed to hypoxia (24 h). ELISA analysis was again used to evaluate PGE_2_ level in CM. This analysis showed that compared to naïve MSCs that were exposed to none (black bar, Fig. 5d), exposure to CM from hypoxic normal HME cells did not induce PGE_2_ secretion from naïve, adipogenic-, osteogenic-, chondrogenic- or fibrogenic fate-adopted MSCs (blue bars, Fig. 5d). In contrast, naïve MSCs and fibrogenic fate-adopted MSCs secreted high levels of PGE_2_ when exposed to CM from hypoxic IRIS291 (orange bars, Fig. 5d), hypoxic IRIS292 (yellow bars, Fig. 5d) or hypoxic IRIS293 (red bars, Fig. 5d) cells. Interestingly, adipogenic-, osteogenic-, and chondrogenic fate-adopted MSCs did not secrete PGE2 when exposed to hypoxic IRIS291 or hypoxic IRIS293 CM. Thus, observations confirm that IRISOE TNBC cell secretome (e.g., IL-6) skews MSC differentiation toward the aggressiveness-promoting PGE_2_-secreting CAF fate, in vitro.

### The opposing role of MSCs toward IRIS-proficient vs. IRIS-deficient TNBC cells

**Fig. 6:**
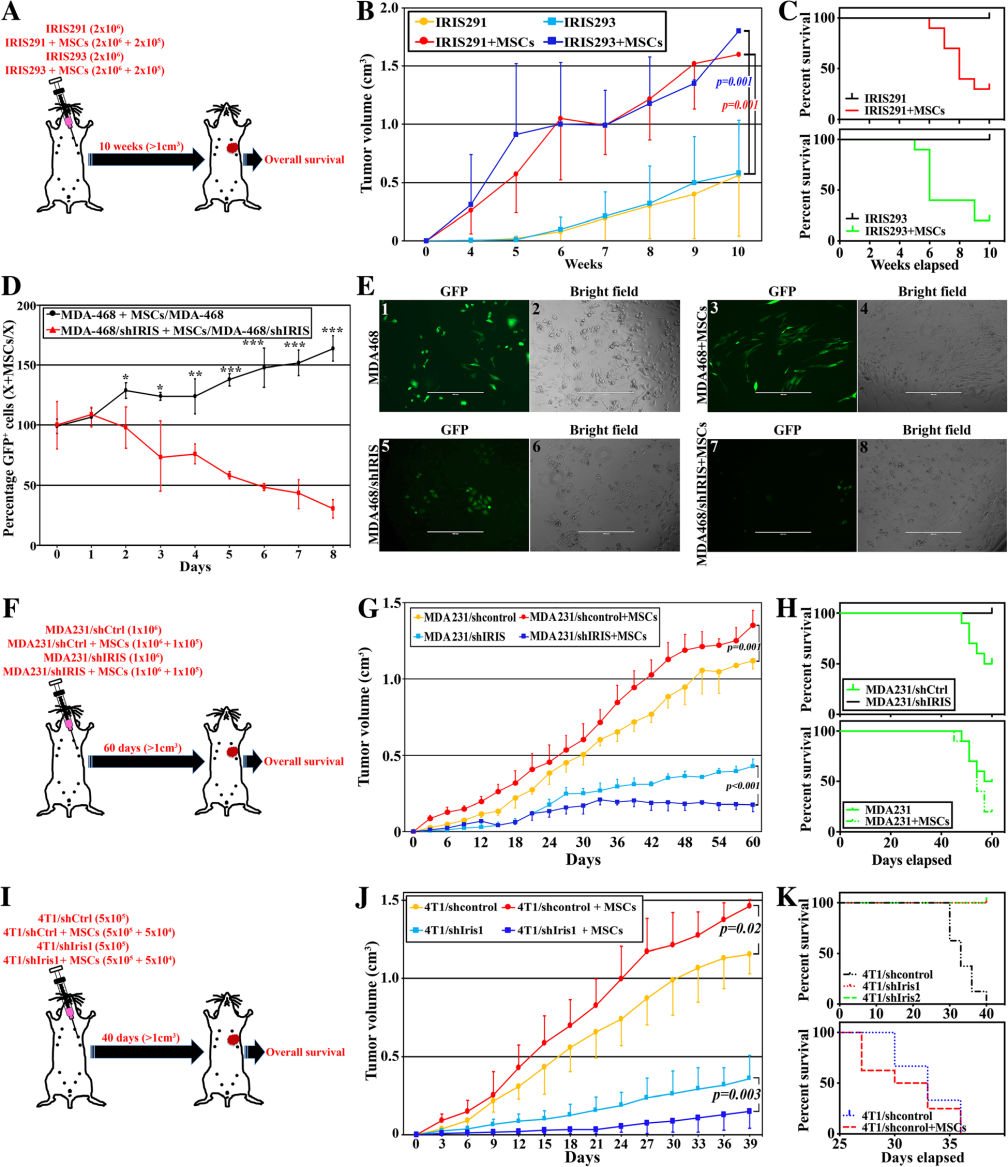
The opposing roles of MSCs in IRISOE TNBC tumors. **a** Schematic representation of the assays presented in (**b**, **c**). **b** Tumor volumes during the 10 weeks following injection in the 2nd thoracic mammary gland of athymic mice of 2 × 10^6^ IRIS291 (yellow), 2 × 10^6^ IRIS291 + 2 × 10^5^ human MSCs (red), 2 × 10^6^ IRIS293 (light blue), or 2 × 10^6^ IRIS293 + 2 × 10^5^ human MSCs (dark blue). **c** Comparison of percentage of survival in athymic mice injected with IRIS291 (upper, black) and IRIS293 alone (lower, black) vs. mice injected with IRIS291 admixed with MSCs (upper, red) or IRIS293 admixed with MSCs (lower, green). **d** Percentage of GFP^+^ cells at days 0–8 in cultures of MDA-468/shCtrl cells + MSCs relative to MDA-468/shCtrl cells (black), and MDA-468/shIRIS cells + MSCs relative to MDA-468/shIRIS cells (red). Representative fluorescence and bright field images of MDA-468/shCtrl (**e**_1 and 2_), MDA-468/shCtrl + MSCs (**e**_3 and 4_), MDA-468/shIRIS (**e**_5 and 6_), or MDA-468/shIRIS + MSCs (**e**_7 and 8_). Assay was done three different times in four replicates. Scale bar = 400 μm. **f** Schematic representation of the assays presented in (**g**, **h**). **g** Tumor volumes during the 60 days following injection of athymic mice 2nd thoracic mammary gland with 1 × 10^6^ MDA-231/shCtrl cells (yellow), 1 × 10^6^ MDA-231/shCtrl cells + 1 × 10^5^ human MSCs (red), 1 × 10^6^ MDA-231/shIRIS cells (light blue), and 1 × 10^6^ MDA-231/shIRIS cells + 1 × 10^5^ human MSCs (dark blue). **h** Upper: comparison of the percentage of survival of mice in **g** injected with MDA-231/shCrtl (green) and MDA-231/shIRIS (black). Lower: comparison of percentage survival of mice in **g** injected with MDA-231/shCtrl admixed with MSCs (dashed) or without MSCs (solid). **i** Schematic representation of the assays presented in (**j**, **k**). **j** Tumor volumes during 40 days following injection of BALB/c mice 2nd thoracic mammary gland with 5 × 10^5^ 4T1/shCtrl cells (yellow), 5 × 10^5^ 4T1/shCtrl cells + 5 × 10^4^ mouse MSCs (red), 5 × 10^5^ 4T1/shIris1 cells (light blue), and 5 × 10^5^ 4T1/shIRIS cells + 1 × 10^4^ mouse MSCs (dark blue). **k** Upper: comparison of the percentage of survival of mice in **g** injected with 4T1/shCrtl cells (black), 4T1/shIris1 cells (red) or 4T1/shIris2 cells (green). Lower: comparison of the percentage of survival of mice in **g** injected with 4T1/shCtrl cells admixed with MSCs (red) or without MSCs (blue)

We previously reported that MSC co-injection triggers faster, bigger, and more aggressive IRISOE TNBC tumors [12]. To explore the effect of IRISOE TNBC cells-MSC bi-directional positive feedback loop on overall survival, athymic female mice were injected in the 2nd thoracic mammary glands with 2 × 10^6^ IRIS291 cells alone (*n* = 10) or admixed with 2 × 10^5^ human MSCs (10:1 ratio, *n* = 10). The second set of mice was injected with 2 × 10^6^ IRIS293 cells alone (*n* = 10) or admixed with 2 × 10^5^ human MSCs (*n* = 10). Mice were followed until death from their disease or until a 10-week time limit (i.e., > 1 cm^3^ tumor volume, Fig. 6a). MSC co-injection promoted an ~ 3-fold increase in IRIS291 (1599.00 ± 436.29 vs. 557.40 ± 519.28, *p = 0.002*, Fig. 6b) and IRIS293 (1799.00 ± 311.11 vs. 580.00 ± 531.04, *p = 0.001*, Fig. 6b) tumor growth. Moreover, while none of the mice injected with IRIS291 cells alone or IRIS293 cells alone died from their tumors by the end of the 10 weeks (Fig. 6c), eight mice injected with IRIS291 + MSCs (Fig. 6c, upper) and seven mice injected with the IRIS293 + MSCs died (Fig. 6c, lower) by the end of the 10 weeks. These outcomes suggest that the IRISOE TNBC cells-MSC bi-directional feedback loop worsens patients’ survival.

We previously noticed that although MDA-468/shIRIS cells can grow in culture, albeit slowly, in vivo they almost completely failed to form tumors [15] (also see Fig. 2b). We reasoned that in vivo, compared to IRIS-proficient TNBC cells, the microenvironment is detrimental to IRIS-deficient TNBC cells. To experimentally define MSC role in the growth potential of IRISOE or IRIS-silenced TNBC cells, we plated 1 × 10^4^ GFP-expressing MDA-468/shCtrl or MDA-468/shIRIS cells alone or with 10 × 10^4^ naïve MSCs for 8 days and cultures were photographed and counted daily. First, starting from the same number of cells, MDA-468/shCtrl cells grew 2-fold during days 1, 2, and 3 after plating compared to MDA-468/shIRIS cells (compare light blue to orange line Additional file 8: Figure S8), at which time the growth of MDA-468/shIRIS cells was enhanced during days 4 and 5 of plating and/or MDA-468/shCtrl cells’ growth was stalled (compare light blue to orange line Additional file 8: Figure S8). However, by day 8, the number of MDA-468/shCtrl cells was 30% higher compared to MDA-468/shIRIS cells (*p = 0.0007*, compare light blue to orange line Additional file 8: Figure S8). More importantly, establishing the percentage of GFP^+^ cells in each culture in the presence vs. absence of MSCs on day 1 as 100%, we noticed gradual enhancement of the growth of MDA-468/shCtrl cells in the presence of MSCs that reached ~ 1.5-fold by day 8 (black line, Fig. 6d and e_1–4_). In contrast, we observed a gradual suppression of the growth of MDA-468/shIRIS by MSCs that reached > 80% by day 8 (red line, Fig. 6d and e_5–8_). So, in low confluence culture, MSCs had a modest effect on IRIS-proficient or deficient TNBC cell growth, while in high confluence culture (i.e., close proximity of the secretome and/or membrane of the two cell types), MSCs had a profound effect on the growth of IRIS-proficient TNBC cells and a detrimental effect on the growth of IRIS-deficient TNBC cells.

To explore this in vivo, we took advantage of the fact that MDA-231/shIRIS formed, albeit small tumors in athymic mice (Fig. 2b). We injected the 2nd thoracic mammary fat pads of athymic mice with 1 × 10^6^ MDA-231/shCtrl (*n* = 10), 1 × 10^6^ MDA-231/shCtrl + 1 × 10^5^ naïve human (h) MSCs (*n* = 10), 1 × 10^6^ MDA-231/shIRIS (*n* = 10), or 1 × 10^6^ MDA-231/shIRIS + 1 × 10^5^ naïve hMSCs (*n* = 10). Tumor volume and overall survival were monitored for 60 days (Fig. 6f). As expected, and previously reported, by day 60 MD-A231/shCtrl were > 250% larger than MDA-231/shIRIS tumors (*p > 0.0000001*, compare yellow to light blue line, Fig. 6g) [12]. Likewise, as previously reported, MSCs enhanced IRISOE TNBC tumor growth [12, 46–48]. Indeed, by day 60, co-injecting MSCs promoted a > 20% increase in MDA-231/shCtrl tumors’ volume (1350.00 ± 100.00 vs. 1119.00 ± 51.63, *p = 0.001*, compare red to yellow lines, Fig. 6g). In contrast, MSCs had a negative effect on the IRIS-deficient TNBC cells. Indeed, by day 60, co-injecting MSCs suppressed MDA-231/shIRIS tumor growth by > 50% (175.60 ± 44.91 vs. 429.40 ± 43.27, *p > 0.0000001*, compare light to dark lines, Fig. 6g).

Additionally, five of the 10 mice injected with MDA-231/shCtrl cells died, whereas none of the mice injected with MDA-231/shIRIS cells died (*p = 0.00001*, Fig. 6h, upper). Moreover, compared to mice injected with MDA-231/shCtrl tumors alone, worse overall survival (OS) was observed in mice co-injected with MDA-231/shCtrl + MSCs (*p = 0.00001*, Fig. 6h, lower). Equally noteworthy, the death occurred several days earlier in the group injected with MDA-231/shCtrl + MSCs compared to those injected with MDA-231 cells alone. These results suggest that IRIS-silencing suppresses TNBC tumors’ growth and aggressiveness. This implies that IRIS inhibition could significantly enhance patients’ overall survival. These data also suggest that the presence of MSCs within the tumor at an earlier stage elevates tumor aggressiveness and can be exploited therapeutically to eliminate the tumor by inhibiting IRIS expression/function. The mechanism of how MSCs kill IRIS-silenced/inactivated cells is still unknown but could perhaps be due to yet unidentified cell-cell contact [49] (and see discussion).

### Further evidence for the opposing role of MSCs toward IRIS-proficient vs. IRIS-deficient TNBC cells

MSC ability to exert an anti-tumor effect as opposed to pro-tumor effect depending on IRIS expression/activity in the tumor was next examined in an immune-competent mouse model. Forty BALB/c mice were injected in the 2nd thoracic mammary gland with 5 × 10^5^ 4T1/shCtrl alone (*n* = 10, Fig. 6i), 5 × 10^5^ 4T1/shCtrl + 5 × 10^4^ mouse (m) MSCs (*n* = 10, Fig. 6i), 5 × 10^5^ 4T1/shIris1 alone (*n* = 10, Fig. 6i), or 5 × 10^5^ 4T1/shIris1 + 5 × 10^4^ mMSCs (*n* = 10, Fig. 6i). By day 40, mice injected with 4T1/shCtrl developed tumors > 3-fold bigger than mice injected with 4T1/shIris1 (1155.20 ± 124.55 vs. 358.60 ± 146.90, *p < 0.0000001*, compare yellow and light blue lines, Fig. 6j). While none of the mice injected with 4T1/shIris1 tumors died by day 40, five of the 10 mice injected with 4T1/shCtrl cells died (Fig. 6k, upper). Identical results were obtained using 4T1/shIris2 cells. Furthermore, injecting naïve mMSCs (10:1 ratio) with Iris-proficient 4T1/shCtrl cells also significantly enhanced tumor growth. Indeed, by day 40, a > 25% increase in the volume of tumors developed in the presence vs. absence of mMSCs was detected (1426.00 ± 41.01 vs. 1155.20 ± 124.55, *p = 0.02*, compare red to the yellow line, Fig. 6j).

Moreover, MSCs induced a worse outcome in these mice. While 5/10 mice died from their disease in the group injected with 4T1/shCtrl cells, 8/10 mice injected with 4T1/shCtrl cells + mMSCs died (Fig. 6k, lower). More importantly, as in the orthotopic model described above in the presence of the hMSCs, mMSCs reduced instead of enhanced the 4T1/shIris1 ability to form tumors. In fact, by day 40, 4T1/shIris1 + mMSC tumors were > 50% smaller than tumors developed using 4T1/shIris1 cells alone (150.60 ± 110.89 vs. 358.60 ± 146.90, *p = 0.003*, compare dark to the light blue line, Fig. 6j). Thus, the data support our conclusion that silencing Iris also converts MSCs within a syngeneic tumor from promoters into suppressors of tumor growth and raises the very interesting possibility that inhibiting IRIS expression and/or activity in patients with established TNBC tumors could trigger death from the inside of the tumors by converting the pro-tumor MSCs into an anti-tumor entity.

### Human data support IL-6/PGE_2_-positive feedback loop enhancing TNBC tumor aggressiveness

**Fig. 7:**
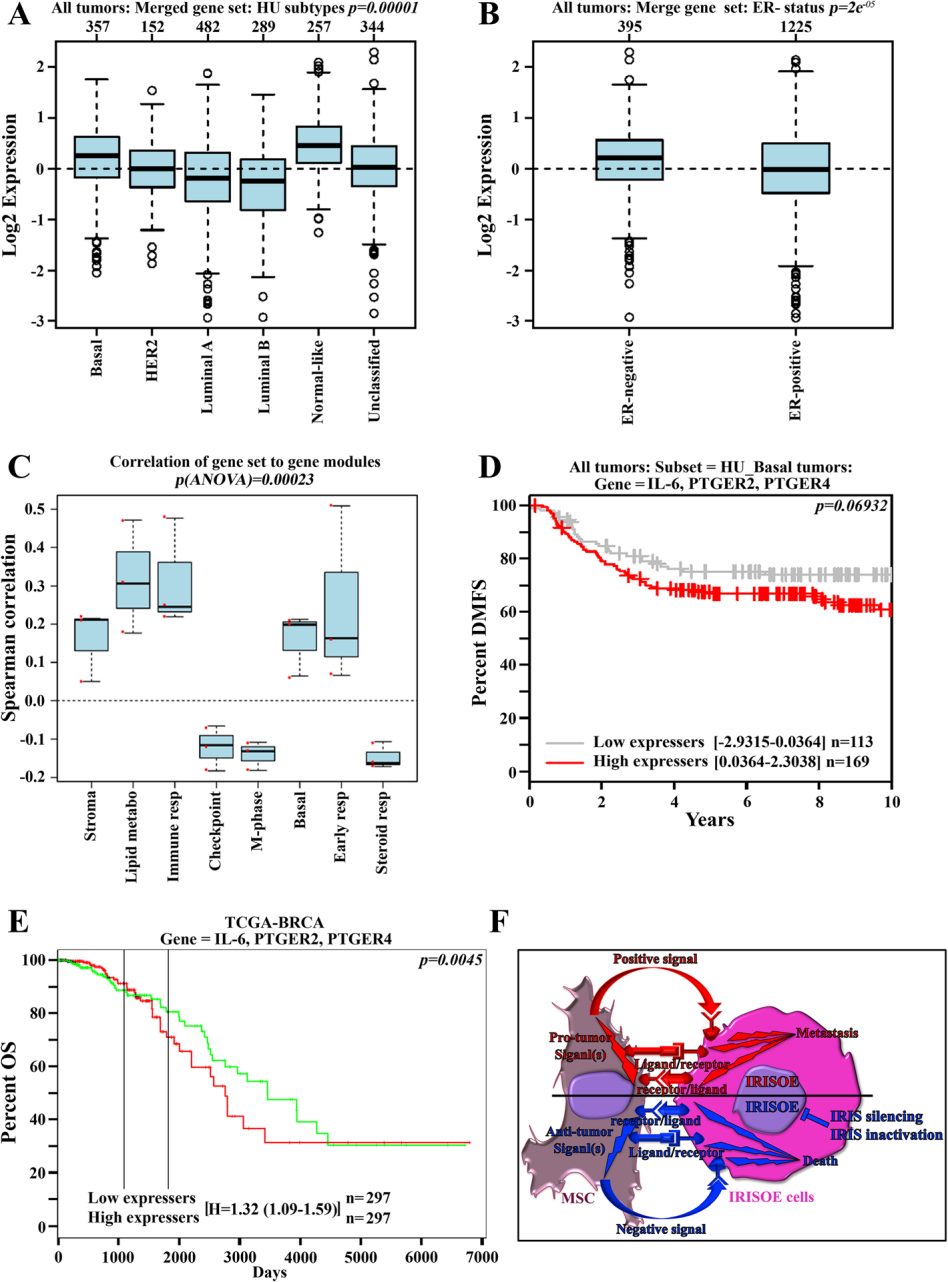
High levels IL6 *plus* PTGER2 *plus* PTGER4 correlate with adverse outcomes in breast cancer patients. **a** Log_2_ expression of IL-6 *plus* PTGER2 *plus* PTGER4 in basal (*n* = 357), HER2 (*n* = 152), luminal A (*n* = 482), luminal B (*n* = 289), normal-like (*n* = 257), and unclassified (*n* = 344) tumors. **b** Log_2_ expression of IL-6 *plus* PTGER2 *plus* PTGER4 in ER-negative (*n* = 395) and ER-positive (n-1225). **c** Spearman correlation of IL-6 *plus* PTGER2 *plus* PTGER4 with the indicated modules (*n* = 1881). Kaplan-Meier analysis of distant metastasis-free survival in basal tumors (*n* = 282), overall survival in BRCA tumors (*n* = 594) of IL-6 *plus* PTGER2 *plus* PTGER4 low expressing (**d**, gray and **e**, green), or high expressing (**d** and **e**, red) breast cancer patients. **f** Overall summary model of the data presented in the current studies and the proposed mechanism for the MSCs pro- vs. anti-IRISOE TNBC tumors roles

So far, the data suggest that high-level IL-6 secreted by TNBC (e.g., IRISOE) cells activates MSCs to secrete PGE_2_ to reciprocally entrain aggressiveness in TNBC cells, most likely within the aggressiveness niche [34]. To validate these data further, we analyzed public databases for the association of the expression of “IL-6, PTGER2, and PTGER4” with aggressive tumor phenotypes. This gene set was significantly associated with the basal subtype (*p = 0.00001*, Fig. 7a) and the ER-negative status in breast cancer samples (*p = 2e*^*−5*^, Fig. 7b). This set also shows positive Spearman correlation with the stromal gene, lipid metabolism gene, immune response gene, basal phenotype gene, and early response gene modules, while showing negative Spearman correlation with the checkpoint gene, M-phase gene, and steroid gene modules in breast cancer samples (*p [ANOVA] = 0.00023*, Fig. 7c).

To evaluate the expression of the gene set “IL-6, PTGER2, and PTGER4” with outcomes in breast cancers, we performed meta-analysis-based biomarker assessment of outcomes in several cohorts of breast cancer patients using Kaplan-Meier plotter (http://kmplot.com [50]). Normalized expression levels of IL-6, PTGER2, and PTGER4 were available for every patient in each cohort; the individual expression levels were summed, and each cohort was then dichotomized into patients with high or low expression of IL-6, PTGER2, and PTGER4 using the median of the summed expression levels in each cohort as the split point. Subsequently, Kaplan-Meier plots and log-rank statistics were calculated to compare the subgroups with high or low expression. Patients with high-level IL-6 *plus* PTGER2 *plus* PTGER4 showed a trend to reduced distant metastasis-free survival (DMSF, *p < 0.06*, Fig. 7d), and a significant reduction in OS (*p = 0.0045*, Fig. 7e), compared to patients with low-level IL-6 *plus* PTGER2 *plus* PTGER4. These data suggest that high expression IL-6 *plus* PTGER2 *plus* PTGER4 in breast cancer could be a diagnostic biomarker for aggressive TNBC tumors (most likely of the IRISOE phenotype) that have a high propensity to metastasize and/or kill the patient. In the near future, we aim to confirm whether or not this signature could stratify IRISOE TNBCs from IRIS-negative TNBCs using prospectively collected tumor samples.

## Discussion

TNBC is a subtype of breast cancer with the poorest clinical outcome that shows strong similarities to BRCA1^mut^ or BRCA1-dysfunctional breast cancers [51]. The poor clinical outcomes and lack of targeted therapy for this subtype warrant a better understanding of the biology underlying the disease in order to develop effective therapeutic strategies against it [51].

IRIS overexpression drives the formation of aggressive TNBC tumors in women or in pre-clinical orthotopic mouse models through promoting the expression of basal biomarkers, EMT inducers, and stemness enforcers, in addition to suppressing the expression of ERα and BRCA1 [14–16, 52]. A large central acellular core of necrosis surrounded by hypoxic and inflamed areas is a common feature of IRISOE TNBC tumors in human or orthotopic models [15, 17]. We recently proposed to name this necrotic/hypoxic/inflamed core “the aggressiveness niche” [34]. Additionally, we argued that TNBC metastatic precursors are born within this niche very early in tumor development [34]. Within this niche, we proposed that the secretome of tumor cells recruit stromal cells including MSCs, tumor-associated macrophages (TAMs), and endothelial cells (ECs) and establish strong bi-directional interactions with them to defend against the unfavorable and harsh conditions within the niche, such as detachment from the ECM, attack by immune cells, growth factor-deprivation, and the hypoxic and inflamed microenvironment.

In a recent publication, we showed that IL-1β secreted by IRISOE TNBC tumor cells within the aggressiveness niche recruited MSCs to the aggressiveness niche and initiated strong bi-directional interactions with them that led to CXCL1 secretion from MSCs. CXCL1 reactivated IRISOE TNBC cells to secrete CCL2 and VEGF that attracted TAMs and ECs, respectively. In the aggressiveness niche, TAMs and ECs secreted S100A8 and IL-8, respectively, that in cooperation with CXCL1 generated metastatic precursors that disseminated from the tumors at an early stage [53]. We argued that these secreted factors could be an independent prognostic factor for low recurrence-free survival (RSF) in IRISOE TNBC patients [54] and showed that Anakinra (the FDA-approved IL-1β inhibitor) alone or together with SB265610 the CXCR2 (the receptor for CXCL1) inhibitor significantly blocked MSC, TAM, and EC recruitment into IRISOE TNBC tumors, reduced the circulating levels of CCL2 and VEGF, tumor growth, and significantly improved mice overall survival.

The present collection of studies supports a second bi-directional interaction “IL-6/PGE_2_” between IRISOE TNBC tumor cells and MSCs in promoting aggressiveness in IRISOE TNBC tumor cells. The fact that naïve MSCs are IL-6R-negative and induced to express the receptor upon exposure to IRISOE TNBC cells IL-6-rich CM is important since it suggests that like Anakinra, IL-6 inhibitors, such as “Tocilizumab,” the monoclonal antibody (mAb) specific for the IL-6 receptor (IL-6R) currently in late phase clinical trials for rheumatoid arthritis [55], is another valid combinatorial drug to be used with IRIS-pep (or drug “in progress”) to treat women with IRISOE TNBC tumors. IL-6 binds to a receptor complex consisting of an 80-kDa ligand-binding chain, known as IL-6R and a 130-kDa signal-transducing chain, named gp130 [56]. Unlike the ubiquitously expressed gp130, IL-6R shows a highly limited expression pattern [57]. IL-6 binds to IL-6R first on target cells, initiating recruitment of gp130 to the complex. IL-6 signaling activates primarily the JAK/STAT3 signaling pathway but can also activate the PI3’K/AKT and MAPK/ERK signaling pathways.

In the steady state, STAT3 resides in the cytoplasm, whereas upon activation, it translocates to the nucleus, mostly as a homodimer to bind a consensus DNA element in target genes, including cyclin D1, Bcl-xL, c-Myc, Mcl1, VEGF, and survivin [58] involved in cancer cell proliferation, differentiation, survival, invasion, inflammation, and immune-suppression functions. Our detailed analysis showing lowered instead of enhanced STAT3 phosphorylation on Y705 in naïve MSCs following exposure to IL-6-rich IRISOE TNBC cells CM (for 24 h as well as 30 min) is at the very least surprising. However, the fact that treatment with rIL-6 (30 ng/ml) also showed the same pattern validates our results. STAT3 possesses two phosphorylation sites that are relevant to function: Y705 and S727 [59]. Traditionally, STAT3^Y705^ phosphorylation is thought to trigger dimerization and activation of the protein. However, recent data have shown that non-phosphorylated STAT3 can activate transcription [60] as a dimer or in complex with other transcription activators, such as IRF1 [61]. STAT3 activity can be exerted by nuclear transcriptional effects, sequestration in signaling endosomes [62], or epigenetically regulated by acetylation on K140 or K685 [63]. Moreover, phosphorylation of S727 alone or in concert with Y705 may also confer STAT3 transcriptional activity [64]. Most importantly, p-STAT3^S727^ intrinsically (not via the negative regulatory loop through SOCS3) regulates the duration of STAT3 activity by promoting dephosphorylation of p-STAT3^Y705^, which shortens the duration of transcriptional activity [65]. The nuclear TC45 phosphatase is the most likely candidate for the p-STAT3^S727^-dependent dephosphorylation of p-STAT3^Y705^. It is possible that TC45 is also upregulated (alternatively activated) in MSCs by IL-6 secreted by IRISOE TNBC cells. This could, at least to some extent, explain the underlying mechanism of our results, and obviously will be the subject of a follow-up study shortly.

Our data also suggest that STAT3 signaling is extremely important for MSC proliferation and migration. In contrast, AKT signaling, while having no effect on MSC proliferation, is important for their migration. Interestingly, ERK signaling, while also having no effect on MSC proliferation, instead blocked MSC migration. Similar data was recently published in different cell types. For example, MAPK/ERK signaling inhibited EGFR/PI3`K/AKT-mediated tissue factor (an initiator of blood coagulation and a participant in cancer progression and metastasis) expression in MDA-MB-231 cells [66]. Moreover, AKT inhibits MAPK’s role in the steroid-induced increase of heart muscle contractility [67]. Finally, inhibiting the AKT pathway, while activating the ERK pathway, contributes to neuronal apoptosis [68]. It is possible that a similar situation also occurs in MSCs exposed to IRISOE TNBC CM, where MAPK/ERK signaling inhibits STAT3 and/or PI3’K/AKT-mediated migration.

Our studies also highlight the role of PGE_2_ in the MSC-enriched microenvironment within the aggressiveness niche in IRISOE TNBC tumors. PGE_2_ activities are mediated through the family of G protein-coupled receptors (EP1-4) that are linked to diverse intracellular signaling pathways [69]. Blocking PGE_2_ synthesis by COX inhibitors, e.g., Celecoxib, reduces tumor metastasis [70]. Thus, blocking EP signaling on tumor cells would also inhibit tumor metastasis [71]. Compared to HME cells, IRISOE TNBC tumor cells express high levels of at least two of these receptors: EP2 and EP4. It is possible that CAFs-secreted PGE_2_ elevates the metastatic ability of IRISOE TNBC cells. This implies an intrinsic aggressiveness-inducing ability of IRISOE TNBC tumor cells [34], which through secreting a high level of IL-6 (and possibly others), recruit and activate MSCs to enhance their own aggressiveness by reciprocally exacerbating EP2 and EP4 activation on tumor cells through PGE_2_. This supports combining IRIS-pep with EP2 and/or EP4 inhibitors and/or Tocilizumab to effectively inhibit the progression of IRISOE TNBC tumors.

Collectively, our data confirm the interesting concept that some tumor types are endowed with an intrinsic ability to promote their own aggressiveness. These tumors, such as IRISOE TNBC secretome, intrinsically activate the microenvironment to reciprocally activate them. We recently proposed that IRISOE TNBC metastatic precursors disseminate from early disease lesions [17]. It is possible that because of the abundant hypoxia and inflammation facing the earliest normal mammary cells, some transform by upregulating IRIS expression and becoming IRISOE TNBC cells. These cells become metastatic precursors through recruiting and activating the microenvironment and disseminate at an early stage. It is thus possible to suggest that using diagnostic tools such as those proposed here could facilitate identifying IRISOE TNBC early disease lesions and treatment with an anti-IRIS combined with an anti-IL-6R and/or anti-EP2/4 inhibitors to block dissemination of metastatic precursors and patients’ death.

Another provocative result from our studies is the fact that naïve MSCs, whether in co-culture or in vivo co-injection assays, have completely opposite roles toward IRIS-proficient vs. IRIS-deficient TNBC cells. It seems that the bi-directional interactions with MSCs in the form of “secreted factors” and/or “cell-cell interactions” (see red Fig. 7f) are beneficial for IRISOE TNBC cells, while detrimental for their IRIS-deficient counterparts (see blue Fig. 7f). It is also possible that loss of IRIS expression/activity in IRISOE TNBC cells converts them to cells that respond in an opposite manner to MSC activity. The more important question is how? We can offer four mutually exclusive and provocative suggestions. All require much more future investigation.

Firstly, we suggest that the secretome of IRIS-deficient TNBC tumor cells converts MSCs from pro-TNBC allies to anti-TNBC cannibalists (perhaps, semi-phagocytes, compare blue to red, Fig. 7f). A negative role for MSCs is not a new observation. Human bone marrow-derived MSCs prevented development of carcinogen-induced lung cancer in a rat model [72], glioblastoma xenografts [73], liver cancer growth [74], inhibited leukemia/lymphoma cell proliferation in vitro and in a mouse model of allogeneic bone marrow transplant [75], and migration and invasion in breast cancer cells [76]. Some proposed that paracrine factors secreted from human or mouse naïve MSCs negatively affect tumor cells through reducing PI3K/AKT activation [74] or secretion of a tissue inhibitor of metalloproteinase-1 and metalloproteinase-2 [76].

Secondly, we suggest that originally MSCs secrete factors (soluble) that could harm IRISOE TNBC cells. Their receptor could be masked in some fashion by IRIS overexpression in TNBC tumor cells. Upon silencing/inactivation of IRIS, this receptor becomes available, exposing the original negative effect of MSCs (compare red and blue Fig. 7f).

Thirdly, our most provocative suggestion is that the masked factor is a membranous rather than a soluble factor. It is possible that IRIS overexpression in TNBC cells inhibits the expression or the surface presentation of ligands/receptors (e.g., CD24 [17]), which might engage with receptor/ligands constitutively expressed by MSCs. The binding of the two would promote TNBC cell death only when IRIS is silenced/inactivated in these cells, and thus, the proposed ligand/receptor is exposed to TNBC cell membranes. Our data (Fig. 6d and e) seem to support the latter suggestion in that we observed the negative effect of MSCs toward IRIS-silenced TNBC cells in highly confluent co-culture rather than sub-confluent cultures (e.g., started on day 2, Fig. 6d and e), i.e., when cell-cell contact becomes more prominent.

Fourth, IRISOE TNBC cells express a receptor that when engaging a ligand on MSC surface inhibits an intrinsic ability of MSCs to be anti-tumor cells. When IRIS is silenced, and the expression of this proposed receptor is decreased, the unmasking of MSC killing ability occurs and tumor cells are attacked and killed. We prefer the last two and are engaged in an intense search for these receptors both on IRISOE (or silenced) TNBC cells and MSCs.

## Conclusions

The bi-directional interactions between BRCA1-IRIS-overexpressing cells and MSCs generate fast growing metastatic TNBC tumors. Suppressing BRCA1-IRIS expression using shRNA or activity using memetic inhibitory peptides blocks TNBC tumor formation and their metastatic ability and significantly prolongs patients’ survival. This was also observed in immunocompetent mice using mouse TNBC tumor cells. We show for the first time an unexpected destabilizing effect for CM from IRISOE TNBC cells to p-STAT3^Y705^ in MSCs with implication for therapies. We also show for the first time an anti-tumor role for MSCs toward IRIS-deficient TNBC cells, also with implication for therapies. For instance, IRIS inactivation triggering the anti-tumor effect of resident MSCs could be pursued as a first-line therapy to combat TNBC tumor formation, progression, and drug-resistant recurrence to significantly reduce TNBC-related mortalities. Finally, the gene set “IL-6 *plus* PTGER2 *plus* PTGER4” once confirmed as a diagnostic tool for detection of IRISOE TNBC tumor cells could activate a treatment protocol directed at blocking the activity of these factors to interrupt IRISOE TNBC tumor cell/MSC-positive bi-directional feedback loop, which could lead to better patient outcomes. Additionally, this gene set could be used as a prognostic tool for the efficacy of this treatment.

## Additional files

Additional file 1: **Figure S1.** Inflammatory cytokine secretion from IRISOE cells. Expression of IL-2, IL-3, IL-4, IL-5, IL-6, and IL-7 in condition media (CM) of HME or IRISOE cells. Assay was performed three separate times. (TIF 1514 kb)

Additional file 2: **Figure S2.** IRISOE induces the TNBC phenotype, while silencing induces the luminal phenotype. The expression of IRIS in HME cells or 3 1° orthotopic IRISOE TNBC tumor cell lines: IRIS291, IRIS292, and IRIS293 (A); in MDA231 and MDA468 cells transfected with siLuc or siIRIS (B); in MDA-231, MD-468, and MDA-453 cells expressing shCtrl or shIRIS (different from the siRNA, C); and in MCF-7 and T47D cells expressing vector or IRIS cDNAs (D). Morphology of normal HME cells (E) compared to IRIS291 (F), IRIS292 (G), and IRIS293 (H) TNBC tumor cells. Morphology of naïve MSCs (I). Morphology of MDA-231/shCtrl (J) compared to MDA-231/shIRIS (K), MDA-453/shCtrl (L) compared to MDA-453/shIRIS (M), and MDA-468/shCtrl (N) compared to MDA-468/shIRIS (O) cells. Morphology of MCF-7/vector (P) compared to MCF-7/IRIS (Q) and T47D/vector (R) compared to T47D/IRIS (S). (TIF 12909 kb)

Additional file 3: **Figure S3.** Normalized mRNA expression of HIF-1α mRNA (A) or protein (B) in HME, IRIS291, IRIS292, and IRIS293 cells expressing siCtrl or siHIF-1α (72 h, *n* = 3). Data obtained in either part using different HIF-1α siRNA. (TIF 1233 kb)

Additional file 4: **Figure S4.** Expression of IRIS in different IRISOE cell clones (A), and p-STAT3Y^705^ in MSCs exposed to CM from these clones for 24 h (B) or 30 min (C). (TIF 4820 kb)

Additional file 5: **Figure S5.** The co-staining or core or periphery of a 1° orthotopic mammary IRISOE TNBC tumor section with CD90 and CD105. Arrows denote red blood cells non-specifically stained with secondary antibodies (TIF 3083 kb)

Additional file 6: **Figure S6.** IRIS-silencing converts TNBC phenotype into luminal phenotype in breast cancer cells. The expression of the luminal biomarker; CK18 expression in shCtrl vs. shIRIS expressing MDA-231 (A and B), MDA-468 (C and D), and MDA-453 (E and F). (TIF 81687 kb)

Additional file 7: **Figure S7.** Representative bright field, oil-red, Alizarin Red S, PicriSirius images of MSC cells exposed to CM from HME (A-D), IRIS291 (E-H), IRIS293 (I-L), MDA-231/shCtrl (M-P), MDA-231/shIRIS (Q-T), MDA-468/shCtrl (U-X), and MDA-468/shIRIS (Y-BB) cells. (TIF 17281 kb)

Additional file 8: **Figure S8.** The growth kinetics of MDA-468/shCtrl cells or MDA-468/shIRIS cell lines grown alone or in the presence of MSCs (1,1) for 8 days. (TIF 1321 kb)
