# Supplementary figures and images for: The pro- and anti-tumor roles of mesenchymal stem cells toward BRCA1-IRIS-overexpressing TNBC cells
Source: Breast Cancer Res. 2019 Apr 24;21:53. doi: 10.1186/s13058-019-1131-2 (PMC6480921; doi:10.1186/s13058-019-1131-2)

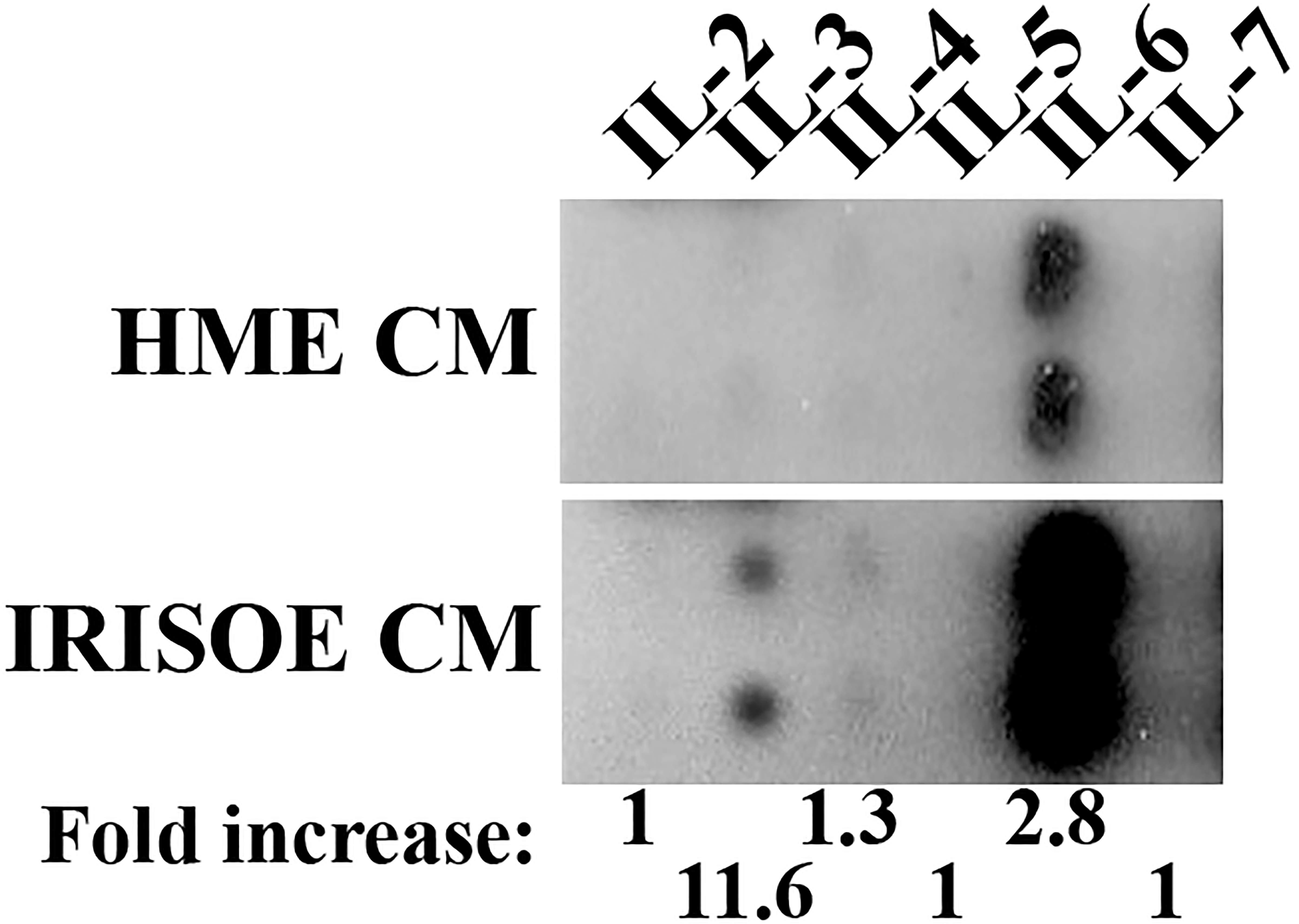

Supplement: Supplementary file 1 — Figure S1. Inflammatory cytokine secretion from IRISOE cells. Expression of IL-2, IL-3, IL-4, IL-5, IL-6, and IL-7 in condition media (CM) of HME or IRISOE cells. Assay was performed three separate times. (TIF 1514 kb) [file 13058_2019_1131_MOESM1_ESM.tif]

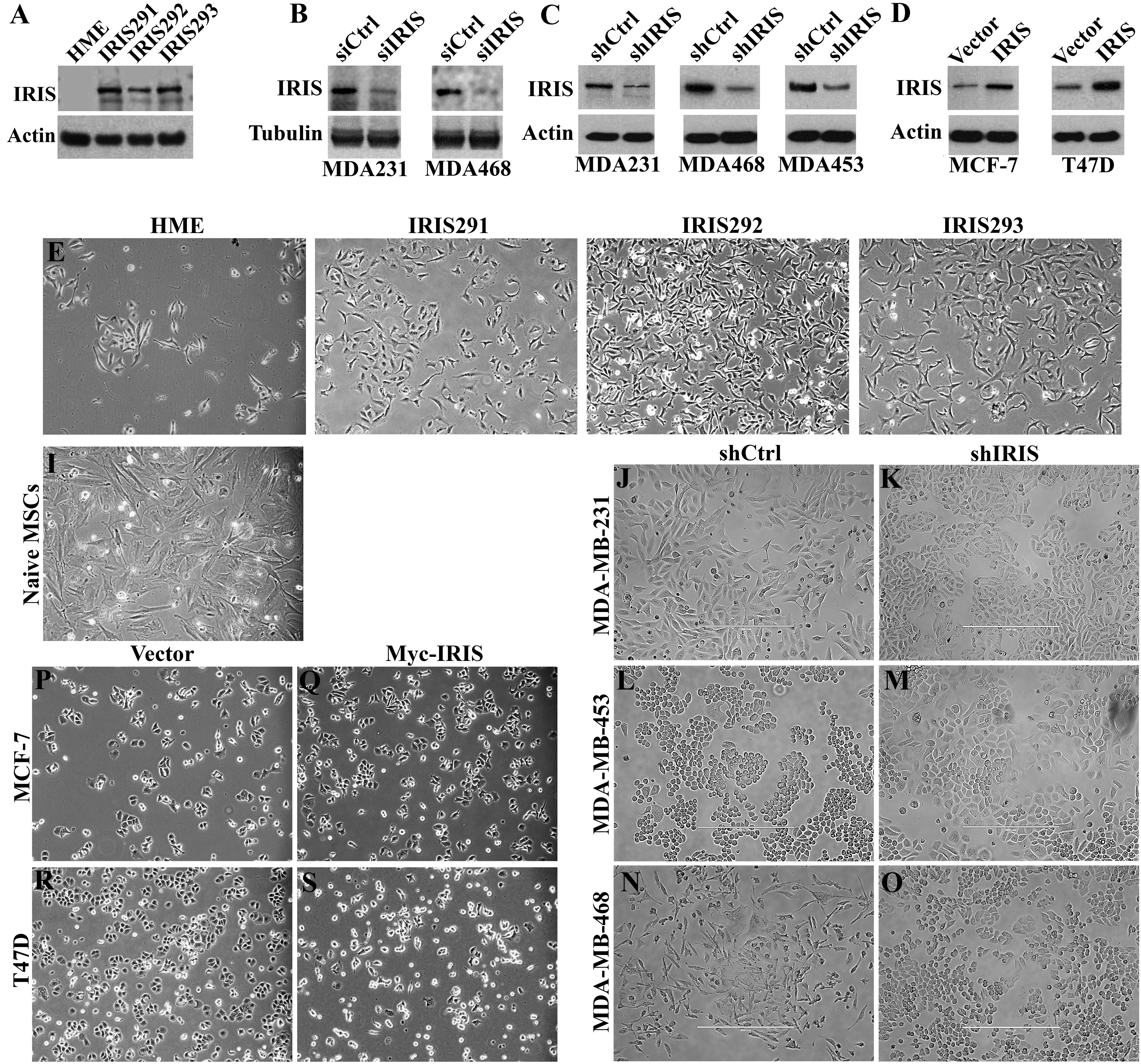

Supplement: Supplementary file 2 — Figure S2. IRISOE induces the TNBC phenotype, while silencing induces the luminal phenotype. The expression of IRIS in HME cells or 3 1° orthotopic IRISOE TNBC tumor cell lines: IRIS291, IRIS292, and IRIS293 (A); in MDA231 and MDA468 cells transfected with siLuc or siIRIS (B); in MDA-231, MD-468, and MDA-453 cells expressing shCtrl or shIRIS (different from the siRNA, C); and in MCF-7 and T47D cells expressing vector or IRIS cDNAs (D). Morphology of normal HME cells (E) compared to IRIS291 (F), IRIS292 (G), and IRIS293 (H) TNBC tumor cells. Morphology of naïve MSCs (I). Morphology of MDA-231/shCtrl (J) compared to MDA-231/shIRIS (K), MDA-453/shCtrl (L) compared to MDA-453/shIRIS (M), and MDA-468/shCtrl (N) compared to MDA-468/shIRIS (O) cells. Morphology of MCF-7/vector (P) compared to MCF-7/IRIS (Q) and T47D/vector (R) compared to T47D/IRIS (S). (TIF 12909 kb) [file 13058_2019_1131_MOESM2_ESM.tif]

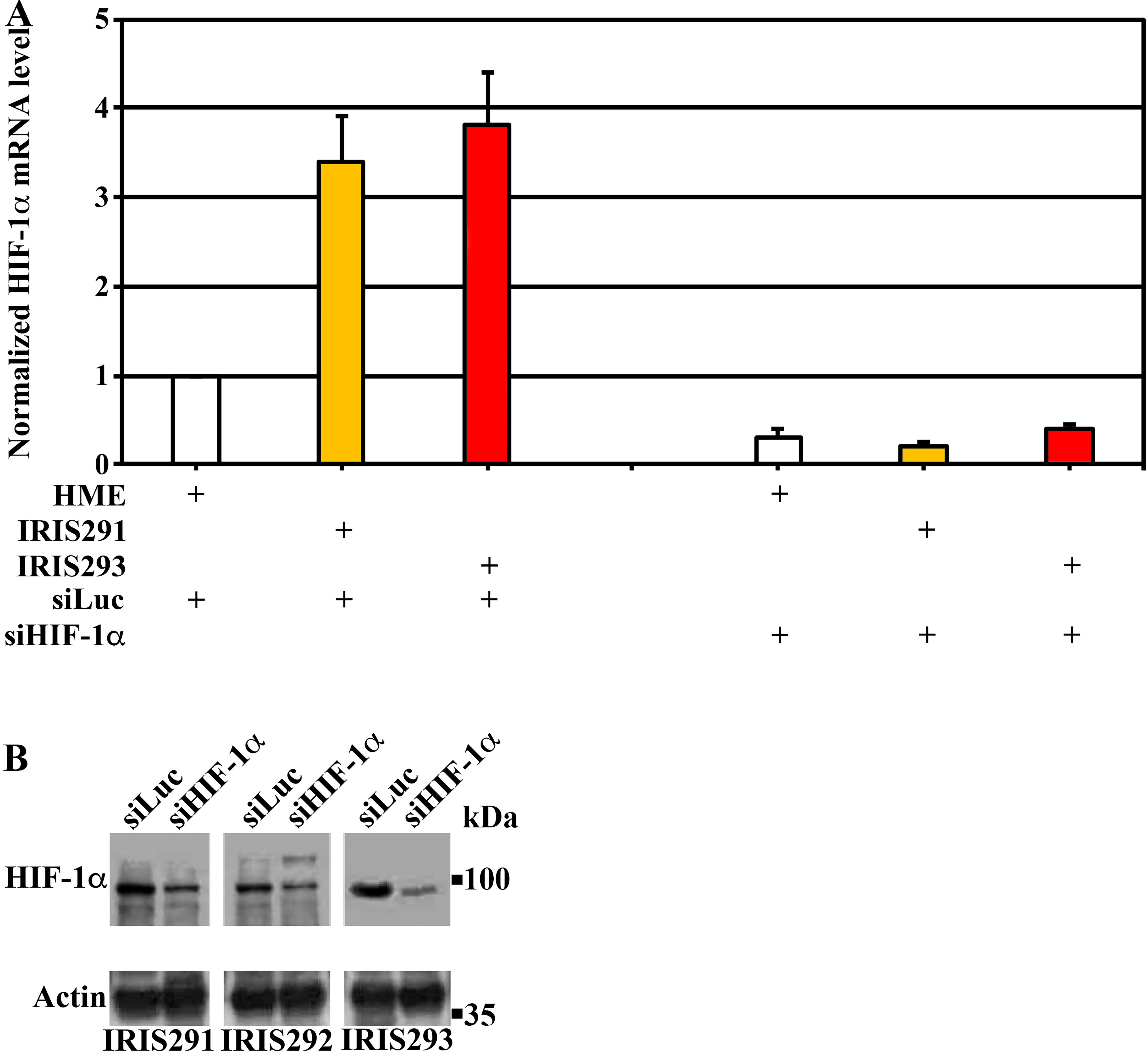

Supplement: Supplementary file 3 — Figure S3. Normalized mRNA expression of HIF-1α mRNA (A) or protein (B) in HME, IRIS291, IRIS292, and IRIS293 cells expressing siCtrl or siHIF-1α (72 h, n = 3). Data obtained in either part using different HIF-1α siRNA. (TIF 1233 kb) [file 13058_2019_1131_MOESM3_ESM.tif]

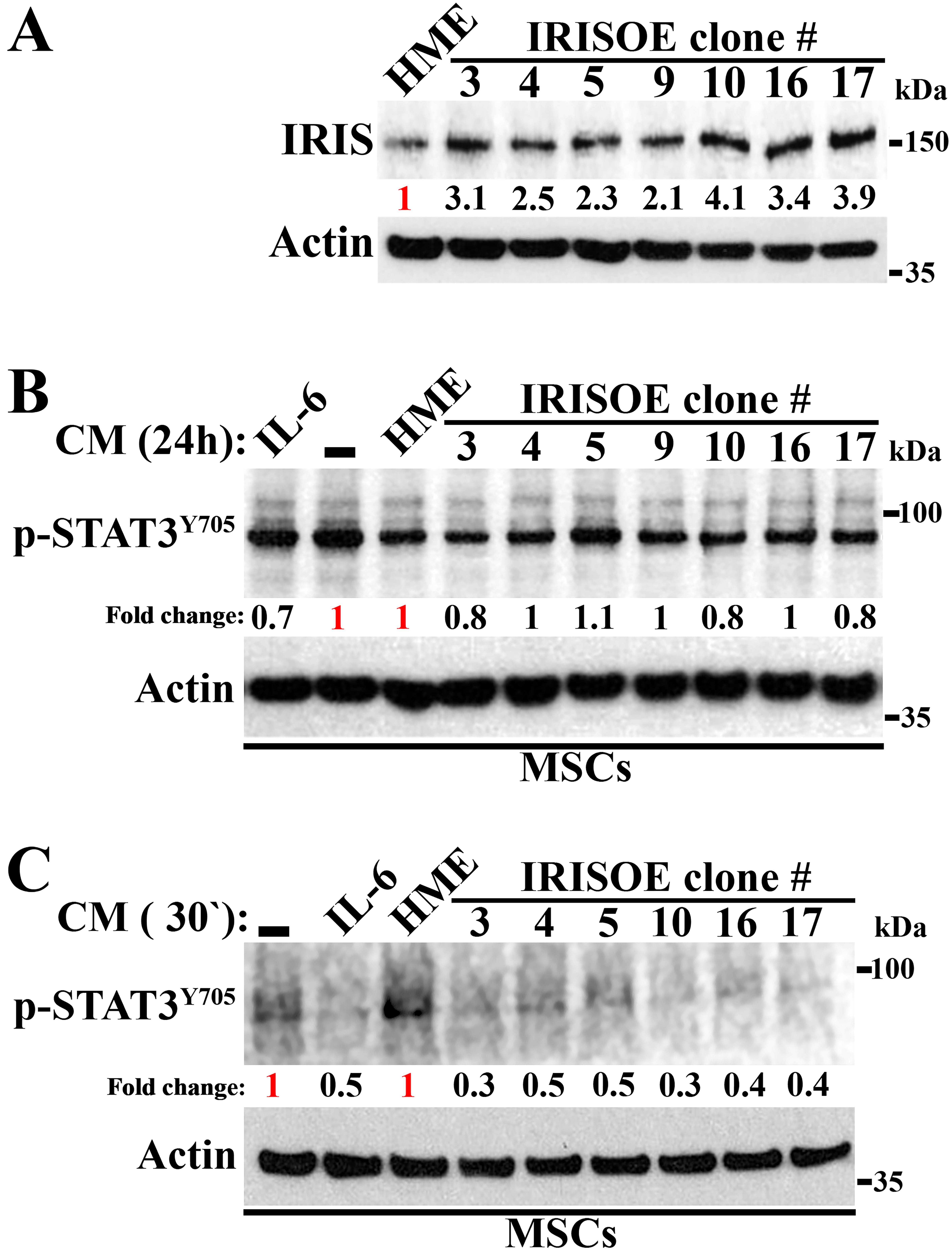

Supplement: Supplementary file 4 — Figure S4. Expression of IRIS in different IRISOE cell clones (A), and p-STAT3Y705 in MSCs exposed to CM from these clones for 24 h (B) or 30 min (C). (TIF 4820 kb) [file 13058_2019_1131_MOESM4_ESM.tif]

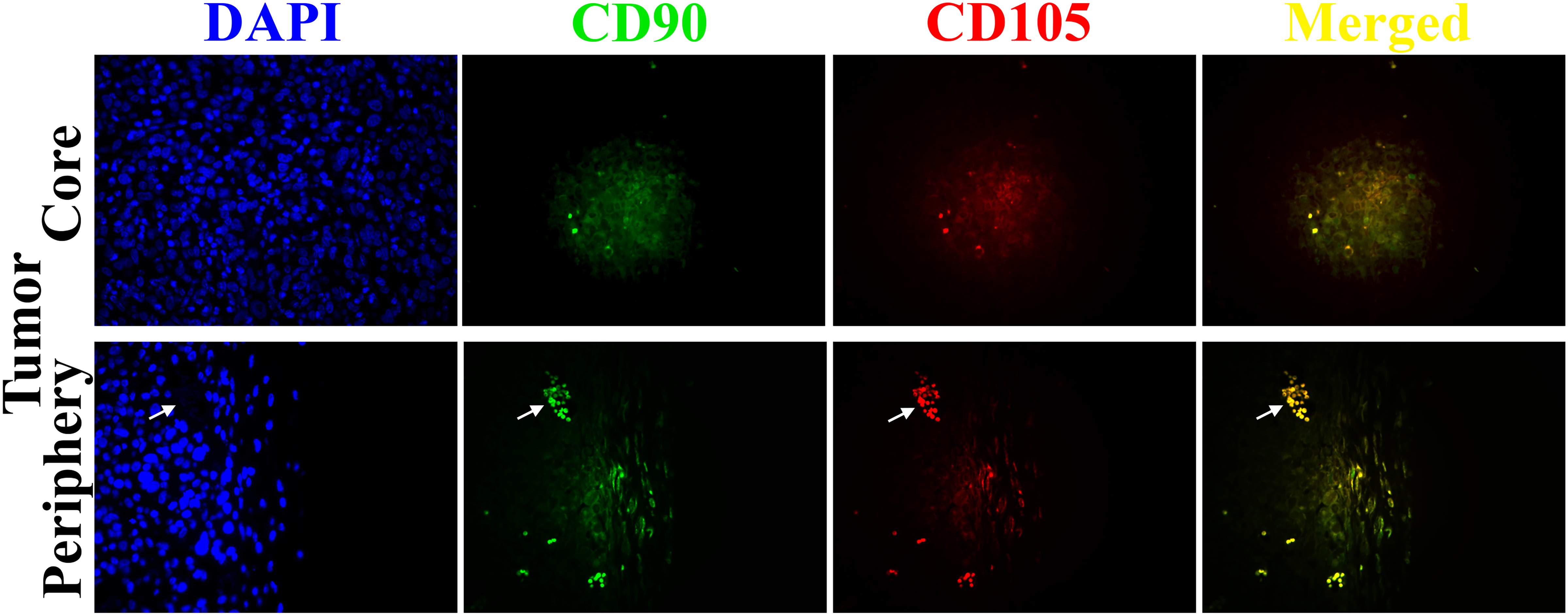

Supplement: Supplementary file 5 — Figure S5. The co-staining or core or periphery of a 1° orthotopic mammary IRISOE TNBC tumor section with CD90 and CD105. Arrows denote red blood cells non-specifically stained with secondary antibodies (TIF 3083 kb) [file 13058_2019_1131_MOESM5_ESM.tif]

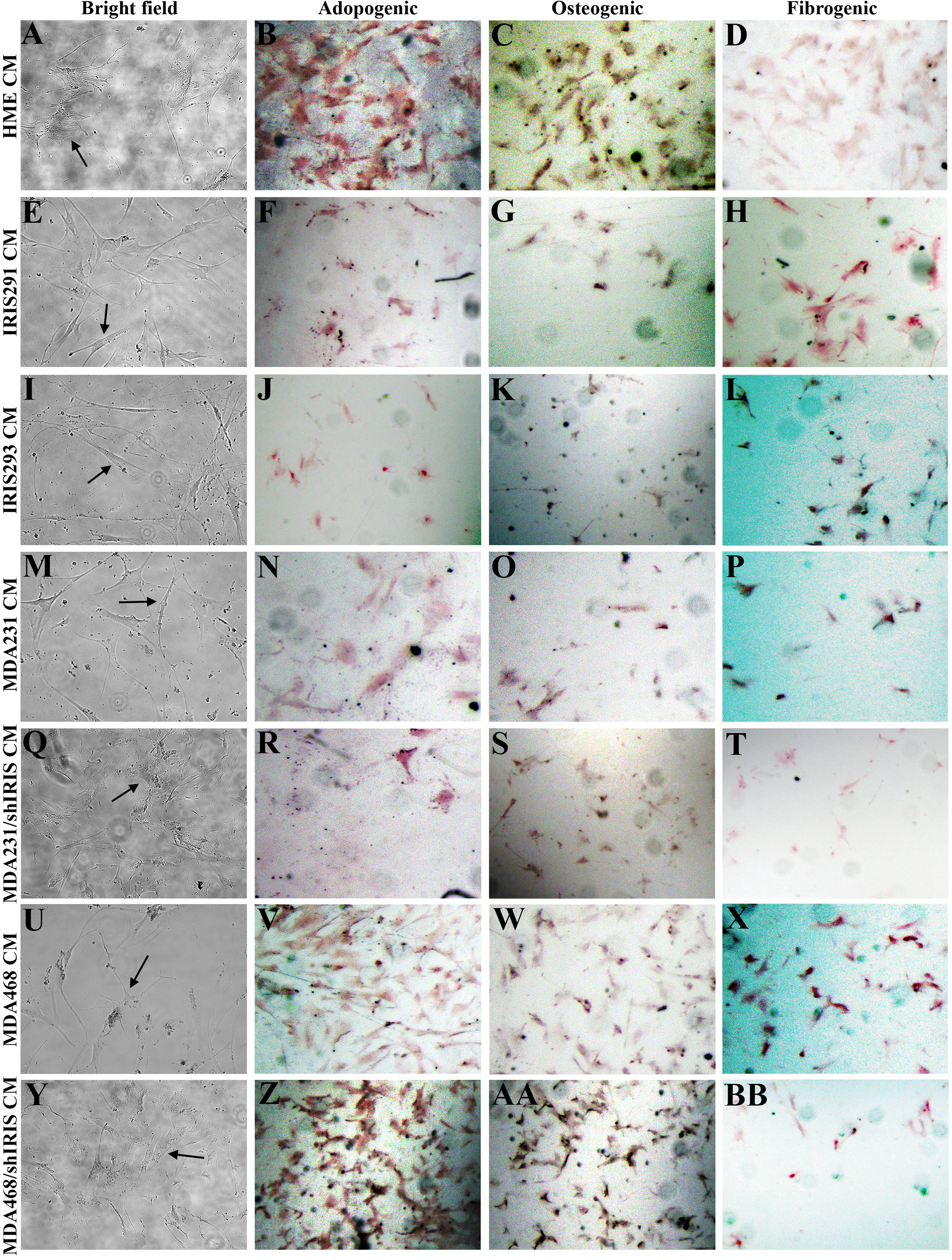

Supplement: Supplementary file 7 — Figure S7. Representative bright field, oil-red, Alizarin Red S, PicriSirius images of MSC cells exposed to CM from HME (A-D), IRIS291 (E-H), IRIS293 (I-L), MDA-231/shCtrl (M-P), MDA-231/shIRIS (Q-T), MDA-468/shCtrl (U-X), and MDA-468/shIRIS (Y-BB) cells. (TIF 17281 kb) [file 13058_2019_1131_MOESM7_ESM.tif]

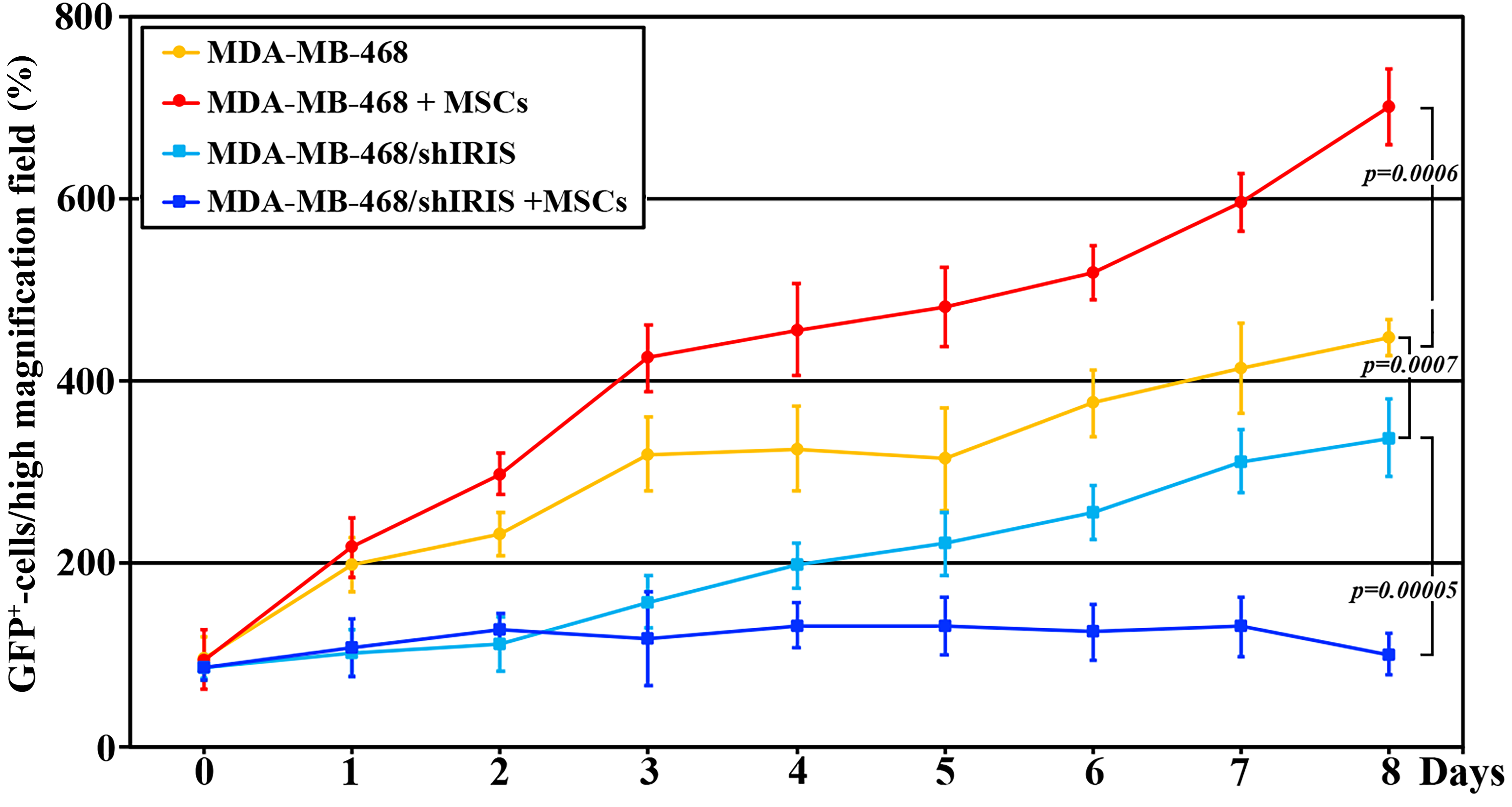

Supplement: Supplementary file 8 — Figure S8. The growth kinetics of MDA-468/shCtrl cells or MDA-468/shIRIS cell lines grown alone or in the presence of MSCs (1,1) for 8 days. (TIF 1321 kb) [file 13058_2019_1131_MOESM8_ESM.tif]
